# Zhuyeqing Liquor Extract Ameliorates Oxidative Stress and Neuroinflammation in D-Galactose-Induced Aging Mice Model

**DOI:** 10.3390/foods15122085

**Published:** 2026-06-09

**Authors:** Ying Han, Lin Wang, Pan Zhen, Xiaoxiao Li, Rong Liu, Hanyue Fu, Xiang Li, Bingye Xu, Fan Wei, Bowei Zhang, Shuo Wang

**Affiliations:** 1 Shanxi XinghuaCun Fen Jiu Distillery Co., Ltd., Fenyang 033000, China; 2 Tianjin Key Laboratory of Food Science and Health, School of Medicine, Nankai University, Tianjin 300071, China

**Keywords:** Zhuyeqing liquor extract, aging, oxidative stress, neuroinflammation

## Abstract

The aging population highlights the urgency of interventions targeting oxidative stress and neuroinflammation. This study investigated the anti-aging potential of Zhuyeqing Liquor Extract (ZLE) using hydrogen peroxide (H_2_O_2_)-induced HT-22 cells and D-galactose-induced aging mice model. Results demonstrated that ZLE exhibited free radical scavenging activity, mitigated neuronal oxidative damage, and suppressed pro-inflammatory cytokine expression. Meanwhile, ZLE alleviated age-related physiological deterioration, improved spatial learning and memory ability, and protected hippocampal neurons in aging mice. Mechanistic analysis revealed that ZLE exerted the bioactivity by activating the SIRT1-Nrf2-HO-1 antioxidant pathway while inhibiting the NF-κB inflammatory pathway. This study suggested that ZLE may have potential as a neuroprotective functional food ingredient, providing a scientific basis for its further development.

## 1. Introduction

With the acceleration of global population aging, age-related degenerative diseases, including Alzheimer’s disease, Parkinson’s disease, and frontotemporal dementia, have gradually become a major public health issue threatening human well-being [1,2]. Therefore, it is necessary to develop safe, effective, and sustainable intervention strategies to delay the aging process and enhance cognitive function. Aging is a complex, multifactorial biological process characterized by a progressive decline in physiological functions. It not only leads to typical neurological symptoms, such as progressive cognitive decline, neuronal damage, and synaptic loss, but also causes a series of systemic adverse effects, including immune dysfunction, organ function decline, metabolic disorders, and increased susceptibility to chronic diseases [3,4].

Among the numerous intrinsic and extrinsic factors regulating the aging process, oxidative stress and neuroinflammation are widely recognized as the core intrinsic mechanisms that drive age-related pathological alterations in the central nervous system (CNS) [5,6]. These two mechanisms interact synergistically and form a vicious cycle that amplifies age-related damage and accelerates the progression of neurodegenerative diseases [7]. The accumulation of reactive oxygen species (ROS) damages key biomolecules, including lipids, proteins, and DNA, thereby triggering mitochondrial dysfunction and apoptosis [8]. These events ultimately lead to neuronal injury and even irreversible neuronal death, which represents a key pathological feature of age-related cognitive decline and neurodegenerative diseases [9,10]. On the other hand, neuroinflammation is a chronic low-grade inflammatory response that spontaneously occurs in the CNS during aging [11]. It is characterized by the activation of astrocytes and microglia in the CNS and the massive secretion of proinflammatory factors [12,13]. These excessive pro-inflammatory factors directly damage neuronal cells and further promote ROS production, thereby exacerbating oxidative stress [14]. Simultaneously, oxidative stress activates the nuclear factor-κB (NF-κB) signaling pathway, which enhances the transcription and release of pro-inflammatory factors, thus establishing a feedback loop between neuroinflammation and oxidative stress [15]. Currently, numerous synthetic anti-aging drugs targeting senescent cells have been developed, but their clinical translation is significantly limited by safety concerns. For example, navitoclax effectively eliminated senescent cells, but it caused thrombocytopenia and neutropenia by inhibiting BCL-2/XL [16]. In addition, metformin has notable limitations, including gastrointestinal adverse reactions, vitamin B12 deficiency, large individual differences in efficacy, and unclear long-term safety [17]. Therefore, researchers are increasingly focusing on exploring multifunctional natural components with antioxidant and anti-inflammatory properties as safer and more promising alternatives.

Zhuyeqing liquor (ZYQ) is a traditional food beverage manufactured using intangible cultural heritage soaking techniques, with Fenjiu as the base liquor and incorporating various medicine and food homology substances, such as *Angelica sinensis* (Oliv.) Diels, *Kaempferia galanga* L., *Lophatherum gracile* Brongn., *Chrysanthemum morifolium* Ramat., *Santalum album* L., *Eugenia caryophyllata* Thunb., *Gardenia jasminoides* Ellis, *Lysimachia capillipes* Hemsl., *Lysimachia foenum-graecum* Hance, *Aucklandia lappa* Decne., *Amomum villosum* Lour., and *Citrus reticulata* Blanco [18]. In recent years, with the in-depth development of the concept of “medicine and food homology”, the application of the plant-derived components in the field of functional foods has attracted increasing attention. Particularly, ZYQ has been included in China’s health food management system and was approved as a health food by the Ministry of Public Health of China in 1998, reflecting its potential value and application prospects in the functional food field. Moreover, the extracts derived from ZYQ have been shown to possess antioxidant activity and exert protective effects by scavenging free radicals [19]. However, systematic research on the active ingredient extracts from ZYQ remains relatively limited at present, especially since the mechanism of anti-aging effects is unclear.

Therefore, the antioxidant and anti-neuroinflammatory effects of Zhuyeqing Liquor Extract (ZLE) were investigated by in vitro cell experiments and in vivo animal experiments. H_2_O_2_-induced HT-22 cells were used to analyze the effects of ZLE on cell viability, oxidative stress activity, and inflammatory factor levels. The D-galactose-induced aging mouse model was established to evaluate the in vivo regulatory effects of ZLE on the physiological status, spatial learning and memory ability, and hippocampal neuron morphology. Furthermore, the molecular mechanisms of the antioxidant and anti-aging effects of ZLE were explored by focusing on the SIRT1-Nrf2-HO-1 and NF-κB signaling pathways. This study aims to provide a scientific basis for the development and utilization of ZLE and offer theoretical support for the transformation of traditional liquor into modern functional foods.

## 2. Materials and Methods

### 2.1. Preparation of ZLE

ZYQ was provided by Shanxi Xinghuacun Fenjiu Distillery Co., Ltd., Fenyang, China. The voucher specimen of ZYQ was preserved at Nankai University (registration number: ZYQ 20240701). ZLE was prepared by removing the ethanol from the liquor via rotary evaporation (EYELA, Tokyo, Japan) at 50 °C, followed by freeze-drying. The obtained ZLE was stored at 4 °C for subsequent experiments, and the corresponding yield was 1.97 g/100 mL.

### 2.2. Macronutrient Quantification and UPLC-Q-Exactive HF-MS Analysis

Total terpenes and total flavonoids were determined via the vanillin–perchloric acid and aluminum nitrate methods, respectively. The chemical profile of ZLE was systematically characterized using UHPLC-MS/MS analysis [20]. Briefly, ZLE was redissolved in 70% methanol and analyzed by Thermo Vanquish UHPLC equipped with Q Exactive HF mass spectrometer (Thermo Fisher Scientific, Waltham, MA, USA). Chromatographic separation was performed on a Zorbax Eclipse C18 column (1.8 μm, 2.1 mm × 100 mm, Agilent Technologies, Santa Clara, CA, USA) at 30 °C with a flow rate of 0.3 mL/min. The injection volume was 2 μL. The mobile phases were 0.1% formic acid (A) and acetonitrile (B). The gradient elution was: 0–2 min, 5% B; 2–6 min, 5–30% B; 6–7 min, 30% B; 7–12 min, 30–78% B; 12–14 min, 78% B; 14–17 min, 78-95% B; 17–20 min, 95% B; 20–21 min, 95–5% B; and 21–25 min, 5% B. The ESI source was run in positive and negative ion modes with the following settings: capillary temperature 330 °C, heater temperature 325 °C, spray voltage 3.5 kV, and S-Lens RF level 55%. Data were collected in DDA mode over m/z 100–1500. Resolutions were 120,000 for full MS and 60,000 for HCD MS^2^ scans. Finally, compounds were identified via matching accurate masses (mass error < 5 ppm) and MS/MS fragment ions against mzCloud and mzVault databases using Compound Discoverer 3.3 (Thermo Fisher Scientific). Detailed information, including retention time, chemical classification, and relative content, is provided in Supplementary Tables S1 and S2.

### 2.3. Antioxidant Activity Measurement

ZLE was formulated into different mass concentrations (1 and 5 mg/mL). All experiments were performed in quadruplicate (*n* = 4). For the blank control, deionized water was used. The standard curves were prepared according to the kit instructions using the provided reference standards. The total antioxidant capacity (Solarbio, Beijing, China, BC1310), superoxide anion radical scavenging capacity (Solarbio, Beijing, China, BC1410), DPPH free radical scavenging capacity (Solarbio, Beijing, China, BC4750), and ABTS^+^ free radical scavenging capacity (Solarbio, Beijing, China, BC4770) were measured following the instructions of the reference kits. The total antioxidant capacity and the scavenging rates of DPPH, ABTS, and superoxide anion radicals were calculated according to the formula provided in the corresponding kit manuals.

### 2.4. Cell Culture

HT-22 cells were cultured in DMEM (Gibco, Grand Island, NY, USA) supplemented with 10% (*v*/*v*) fetal bovine serum (FBS, Gibco, Grand Island, NY, USA) and 1% penicillin-streptomycin double antibody and maintained in an incubator at 37 °C with 5% CO_2_. The culture medium was replaced every 24–48 h, and cells were passaged when reaching 80% to 90% confluence. Finally, the logarithmic growth phase of HT-22 was selected for subsequent experiments.

### 2.5. Cell Viability Measurement

The CCK-8 assay was used to measure cell viability. HT-22 cells were seeded into 96-well plates at a density of 5 × 10^3^ cells/well. After incubation for 24 h, the culture medium was discarded. The fresh medium containing ZLE at different concentrations (10, 25, 50, 100 and 500 μg/mL) was added to the experimental wells. The control group was treated with fresh medium without ZLE. After incubation for 24 h, 10 μL CCK-8 reagent was added to the cells and incubated at 37 °C for 1 h. The OD value at 450 nm was measured using a microplate reader (Tecan, Infinite M200 Pro, Männedorf, Switzerland).

### 2.6. Half-Maximal Inhibitory Concentration (IC_50_)

HT-22 cells were seeded at a density of 5 × 10^3^ cells/well into 96-well plates. The experimental group was supplemented with fresh medium containing different concentrations of H_2_O_2_ (200 μM, 400 μM, 600 μM, 800 μM and 1000 μM). After 4 h of treatment, 10 μL CCK-8 reagent was added to each well, and the plates were incubated at 37 °C. The OD value at 450 nm was measured using a microplate reader (Tecan, Infinite M200 Pro, Switzerland). Then, the IC_50_ value was calculated by plotting a dose–response curve using GraphPad Prism 10.1.1.

### 2.7. Determination of the Effects of ZLE on H_2_O_2_-Induced HT-22 Cells

Based on the results of Section 2.6, the H_2_O_2_ concentration (650 μM) was selected for treating HT-22 cells in subsequent experiments. The cells were divided into a control group, a H_2_O_2_-induced model group, and a ZLE intervention group. The control and model groups were cultured in standard medium, while the ZLE treatment group was cultured in fresh medium containing different concentrations of ZLE (10, 50, 100, and 500 μg/mL). After 24 h of ZLE pretreatment, the medium was discarded. Subsequently, the control group was cultured in standard medium without H_2_O_2_, while the model group and all ZLE-treated groups were cultured in fresh medium containing H_2_O_2_ to induce oxidative stress. All cells were incubated for 4 h at 37 °C with 5% CO_2_. Finally, cell viability was assessed using CCK-8 as described in Section 2.5.

### 2.8. ROS Reduction Assay

Cells were pre-treated with ZLE (10 and 100 μg/mL) and subsequently exposed to 650 μM H_2_O_2_ to induce oxidative stress. The control (Ctrl) group was treated with fresh medium without H_2_O_2_ or ZLE, while the model (H_2_O_2_) group was treated with 650 μM H_2_O_2_. Following trypsin digestion and collection, the cells were resuspended in 10 μM DCFH-DA and incubated at 37 °C in the dark for 20 min. Subsequently, cells were washed with DEME medium three times to remove excess DCFH-DA. Fluorescence intensity was recorded at an excitation wavelength of 488 nm and an emission wavelength of 525 nm.

### 2.9. Cell Cycle Measurement

HT-22 cells were treated as described in Section 2.8. Following treatment, cells were collected, washed with 1 mL ice-cold PBS, and then fixed in 1 mL ice-cold 70% (*v*/*v*) ethanol at 4 °C for 24 h. Then, cells were stained by incubating with propidium iodide (PI) staining solution at 37 °C in the dark for 30 min. Finally, cell cycle distribution was analyzed using flow cytometry (FACSymphony A1, BD Biosciences, San Jose, CA, USA).

### 2.10. Animal Experiments

The animal experiments were designed as in a previous study with some modifications [21,22,23]. The male ICR mice (8 weeks old) were purchased from Beijing Vital River Laboratory Animal Technology Co., Ltd. (Beijing, China) and reared in the SPF-grade barrier environment. The animal experiments were approved by the guidelines of the Animal Care and Use Committee of Nankai University (Permit Number: SYXK (Jin) 2019-0001). All mice were allowed free access to food and water for 1 week under conditions at 22 ± 2 °C and a relative humidity of 55 ± 15% with a 12 h light/dark cycle. Then, the mice were divided randomly into five groups (*n* = 8): Control group (CON), Model group (MOD), Vitamin C group (VC), Low-dose ZLE group (ZL), and High-dose ZLE group (ZH). Here, mice in the MOD, VC, ZL, and ZH groups were subcutaneously injected with 500 mg/kg D-galactose and orally administered with distilled water, 100 mg/kg VC solution, 100 mg/kg ZLE, and 200 mg/kg ZLE, respectively. The mice in the CON group were subcutaneously injected with physiological saline and orally administered with distilled water daily. The subcutaneous injection and oral administration were continued for 8 weeks. The change in body weight and food intake of mice was recorded every week. The mice were anesthetized with isoflurane and euthanized by cervical dislocation at the end of the intervention. The serum was obtained by centrifuging at 3000 *g* for 15 min at 4 °C. The weight of the spleen and liver was measured. The whole brain tissue was divided into two parts: one part was fixed in 4% paraformaldehyde for histological examination, and the other part was rapidly stored at −80 °C until analysis.

### 2.11. Morris Water Maze (MWM)

The MWM test was measured as the previously reported method of Vorhees et al. with slight modifications [24]. MWM tests were performed by researchers blinded to the animal groups. The MWM system consists of a circular pool with a platform and is equipped with the Morris visual tracking system (Tracking Master-V4.0-MWM, Fanbi Intelligent Technology Co., Ltd., Shanghai, China). The water temperature of the pool was maintained at 22 ± 1 °C throughout the experiment. The hidden platform was placed in the center of the southwest quadrant, and the water pool was filled to a level 1 cm above the platform. Four distinct visual markers with different shapes and colors were adhered to the inner walls of the four quadrants, respectively. The swimming activity of mice was recorded via an overhead camera. The entire test included a five-day training trial and a one-day probe trial. During the training trial, four trials were conducted per day with at least a 30 min interval between consecutive trials. In each trial, mice were placed facing the pool wall from each of the four quadrants to find the platform and remain for 10 s. If the mice failed to find the platform within 60 s, they were guided to the platform and kept for 10 s. The escape latency (s) was recorded. During the probe trial, the platform was removed. All mice were placed in the quadrant opposite to the target quadrant and allowed to swim freely for 60 s. The trajectory diagram, the time spent in the target quadrant, and the number of platform crossings were recorded by the tracking system for subsequent statistical analysis.

### 2.12. Biochemical Assays

The levels of Aspartate aminotransferase (AST, C010-2-1), Alanine aminotransferase (ALT, C009-2-1), Creatinine (Cre, C011-2-1), Blood urea nitrogen (BUN, C013-3-1), Malondialdehyde (MDA, A003-1-2), Glutathione (GSH, A006-2-1) and Glutathione peroxidase (GSH-PX, A005-1-2) and the activity of superoxide dismutase (SOD, A001-3-2) in serum were measured using commercially available kits (Nanjing Jiancheng Bioengineering Institute, Nanjing, China). All experimental procedures were performed according to the manufacturers’ protocols and were measured using a microplate reader (Tecan, Infinite M200 Pro, Männedorf, Switzerland).

### 2.13. Hematoxylin and Eosin (H&E) Staining

The brain tissues were collected and fixed in paraformaldehyde (4%). The samples were dehydrated in ethanol, embedded with paraffin and sliced. Then the tissue sections were deparaffinized in xylene and stained with H&E. Finally, the sections were mounted with neutral resin and covered with a coverslip.

### 2.14. Immunofluorescence (IF) Staining

The IF staining was performed by the previous method with some modifications [25]. The mouse brain sections were dewaxed, rehydrated, and subjected to antigen retrieval by heating in EDTA. After washing in PBS, sections were blocked with goat serum at room temperature for 30 min. The blocking solution was discarded, and the antibodies GFAP (HUABIO, ET1601-23, 1:500) and Iba1 (HUABIO, ET1705-78, 1:500) were added to the sections, which were incubated overnight at 4 °C in a humidified box with adequate water supplement to prevent antibody evaporation. Subsequently, the sections were washed and incubated with fluorescent secondary antibody (goat anti-rabbit IgG/594: Boster, BA1142, 1:200; goat anti-rabbit IgG/488: Beyotime, A0562, 1:200) at room temperature in the dark for 50 min. After PBS washes, DAPI (Solarbio, Beijing, China, C0065) was added to the sections and incubated at room temperature for 2 min. Finally, the sections were mounted with anti-fluorescence quenching medium, and the fluorescence of target proteins (GFAP and Iba1) and nuclei in the hippocampus was visualized under a fluorescence microscope (3DHISTECH, Pannoramic Scan, Budapest, Hungary). Image analysis was performed using SlideViewer 2.5, and the positive area (%) was calculated by ImageJ 2.14.0.

### 2.15. Reverse-Transcription Quantitative PCR (RT-qPCR) Analysis

The relative mRNA expression levels of tumor necrosis factor-α (TNF-α), interleukin-1β (IL-1β), interleukin-6 (IL-6), catalase (CAT), superoxide dismutase (SOD), nuclear factor-κB (NF-κB), sirtuin 1 (SIRT1), nuclear related factor-2 (Nrf2), heme oxygenase-1 (HO-1), and β-actin were analyzed using RT-qPCR according to previous methods with several modifications [26]. Briefly, the HT-22 cells and brain tissues were homogenized with TRIzol reagent, and total RNA was extracted. The RNA was assessed by a NanoDrop and synthesized into cDNA (R433, Vazyme, Nanjing, China). Then, the cDNA was mixed with gene-specific primers (Table A1) and amplified using SYBR qPCR master mix (Q711, Vazyme, Nanjing, China). The thermal cycling conditions were as follows: an initial denaturation at 95 °C for 30 s, followed by 40 cycles of denaturation at 95 °C for 5 s and annealing at 60 °C for 30 s. The relative mRNA expression levels were calculated by the 2^−ΔΔCT^ method, with β-actin serving as the internal reference.

### 2.16. Western Blot Analysis

20–30 mg of brain tissue was homogenized in 200 μL lysis buffer for 40 min. The mixture was centrifuged at 12,000 *g* for 10 min, and the supernatant was collected for protein quantification using the BCA assay. Western blot analysis was conducted by the previous study with some modifications [27]. Briefly, the primary antibodies against NF-κB p-P65 (1:800), NF-κB P65 (1:3000), β-actin (1:10,000), and HRP-conjugated secondary antibody (1:10,000) were used. The protein bands were visualized using the Pierce ECL Western Blot Kit (Thermo Fisher Scientific, Waltham, MA, USA) and captured with a gel imaging system (Bio-Rad, Hercules, CA, USA). The corresponding gray values were calculated by ImageJ software (version 2.14.0).

### 2.17. Statistical Analysis

All image quantification and data analysis were completed in a blinded manner. All data was presented as mean ± standard deviation (SD). Statistical analysis was conducted using SPSS 27.0 and Prism 10.1.1 software. Student’s t-test was used to compare differences between two groups. One-way ANOVA with Tukey’s test was performed to compare differences among more than two groups. Two-way repeated-measures ANOVA with Tukey’s test was carried out to analyze escape latency in the Morris water maze. All differences with *p* < 0.05 were considered statistically significant.

## 3. Results

### 3.1. Phytochemical Characterization of ZLE

To further clarify the bioactive constituents of ZLE, its chemical profile was characterized. Quantitative analysis showed that the total terpene and total flavonoid contents were 2.841 ± 0.039 and 1.098 ± 0.008 mg/mL, respectively. UPLC-Q-Exactive HF-MS analysis identified a total of 389 phytochemical components in ZLE, mainly including iridoid glycosides, organic acids, and flavonoids (Figure S1 and Tables S1 and S2).

### 3.2. Antioxidant Effects of ZLE

**Figure 1:**
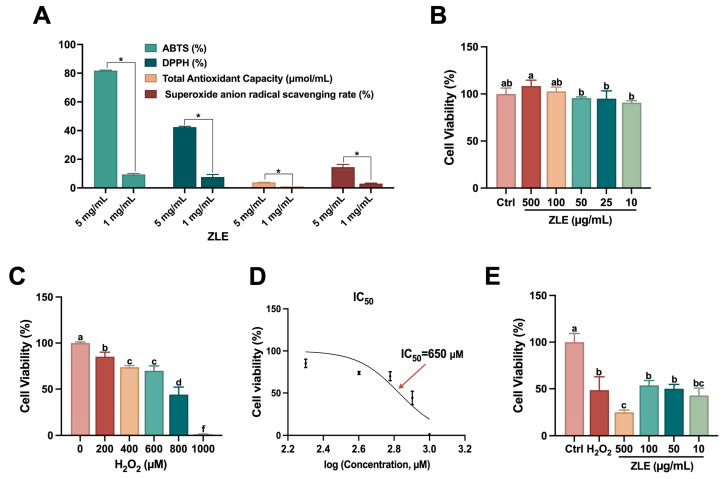
(**A**) The antioxidant effects of ZLE, * *p* < 0.05. (**B**) The effect of the different doses of ZLE on the cell viability of HT-22 cells. (**C**,**D**) The half-maximal inhibitory concentration (IC_50_) of H_2_O_2_-induced cytotoxicity in HT-22 cells. (**E**) The protective effects of different doses of ZLE on H_2_O_2_-induced cytotoxicity in HT-22 cells. Different lowercase letters denote statistically significant differences (*p* < 0.05) between groups.

As shown in Figure 1A, when the concentration of the ZLE ranged from 1 to 5 mg/mL, the total antioxidant activity varied from 1.06 to 3.84 µmol/mL. At a ZLE concentration of 5 mg/mL, the radical scavenging rates of superoxide anion, DPPH, and ABTS free radicals were 14.3%, 42.4%, and 81.8%, respectively. In contrast, the corresponding values were 3.1%, 7.6%, and 9.4% at 1 mg/mL. Therefore, the ZLE exhibited concentration-dependent antioxidant effects, with higher concentrations producing stronger activity.

### 3.3. Effects of ZLE on the Cell Viability of H_2_O_2_-Induced HT-22 Cells

To further investigate the protective effects of the ZLE against oxidative stress in neuronal cells, an H_2_O_2_-induced oxidative stress model was established in HT-22 cells. Preliminary results showed that the ZLE at concentrations of 100 μg/mL and 500 μg/mL promoted the proliferation of HT-22 cells (Figure 1B). Additionally, the IC_50_ of H_2_O_2_-induced cytotoxicity in HT-22 cells was 650 μM (Figure 1C,D). Based on this, the regulatory effects of ZLE at different concentrations on the cell viability of H_2_O_2_-induced HT-22 cells were further explored. The results showed that the survival rate of HT-22 cells treated with H_2_O_2_ was significantly decreased at 500 μg/mL ZLE, while ZLE treatments at 50 μg/mL and 100 μg/mL slightly enhanced the cell survival rate (Figure 1E). Notably, ZLE at high concentration (500 μg/mL) exhibited potential toxicity in the H_2_O_2_ model, while low to medium concentrations (50 μg/mL, 100 μg/mL) exerted a protective effect against oxidative stress. These findings suggested that the modulatory effects of ZLE on HT-22 cells displayed protective activity and dose-dependent toxicity.

### 3.4. Effects of ZLE on Oxidative Stress and Inflammatory Levels in H_2_O_2_-Induced HT-22 Cells

**Figure 2:**
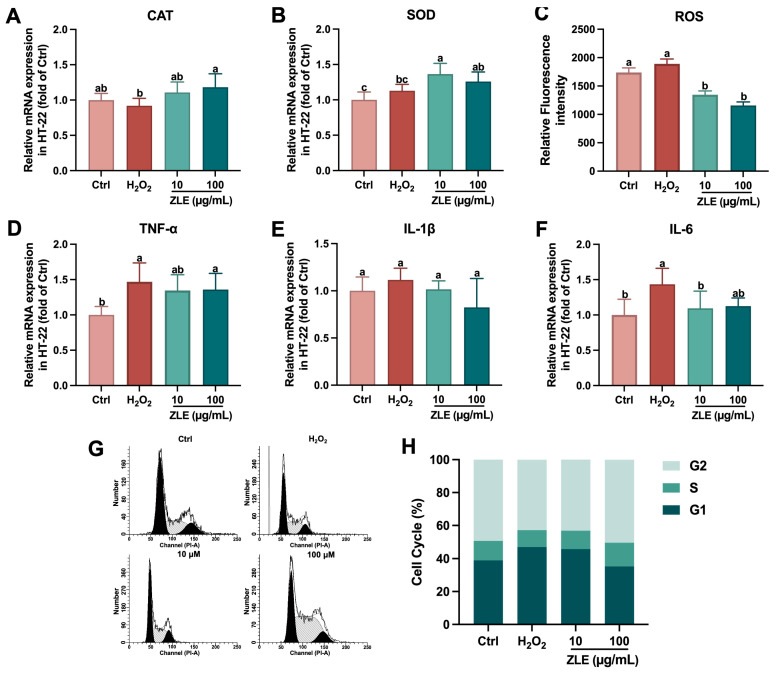
Effects of different doses of ZLE on the levels of CAT (**A**), SOD (**B**), ROS (**C**), TNF-α (**D**), IL-1β (**E**), IL-6 (**F**), and cell cycle (**G**,**H**) in H_2_O_2_-induced HT-22 cells. Different lowercase letters denote statistically significant differences (*p* < 0.05) between groups.

The regulatory effects of ZLE on major antioxidant enzymes and pro-inflammatory cytokines in H_2_O_2_-stimulated HT-22 cells were explored by RT-qPCR. As shown in Figure 2A,B, the relative mRNA expression level of CAT was markedly elevated in HT-22 cells treated with 100 μg/mL ZLE (*p* = 0.019), whereas the relative mRNA expression level of SOD was significantly up-regulated after intervention with 10 μg/mL ZLE (*p* = 0.018). In addition, the ROS levels in H_2_O_2_-induced HT-22 cells were significantly reduced following treatment with 10 μg/mL and 100 μg/mL ZLE (*p* < 0.01) (Figure 2C). Notably, H_2_O_2_-induced oxidative stress further triggered an inflammatory response in HT-22 cells, which was characterized by a significant increase in the relative mRNA expression levels of the pro-inflammatory cytokines TNF-α and IL-6 (Figure 2D–F). Compared with the H_2_O_2_-induced model group, the ZLE intervention presented a downward trend in the relative expression levels of TNF-α, IL-1β and IL-6 in HT-22 cells, indicating ZLE exerted potential anti-inflammatory effects against H_2_O_2_-induced neuroinflammation in HT-22 cells.

### 3.5. Effects of ZLE on the Cell Cycle of H_2_O_2_-Induced HT-22 Cells

The regulatory effects of the ZLE on the cell cycle progression in H_2_O_2_-induced HT-22 cells were investigated by using 10 μg/mL and 100 μg/mL ZLE (Figure 2G,H). The increase in G1 phase and decrease in G2 phase demonstrated that oxidative stress induced cell cycle arrest in HT-22 cells. Notably, the disruptions of the HT-22 cell cycle were alleviated following intervention with 100 μg/mL ZLE.

### 3.6. ZLE Regulated the Growth and Physiological Indices of D-Galactose-Induced Aging Mice

**Figure 3:**
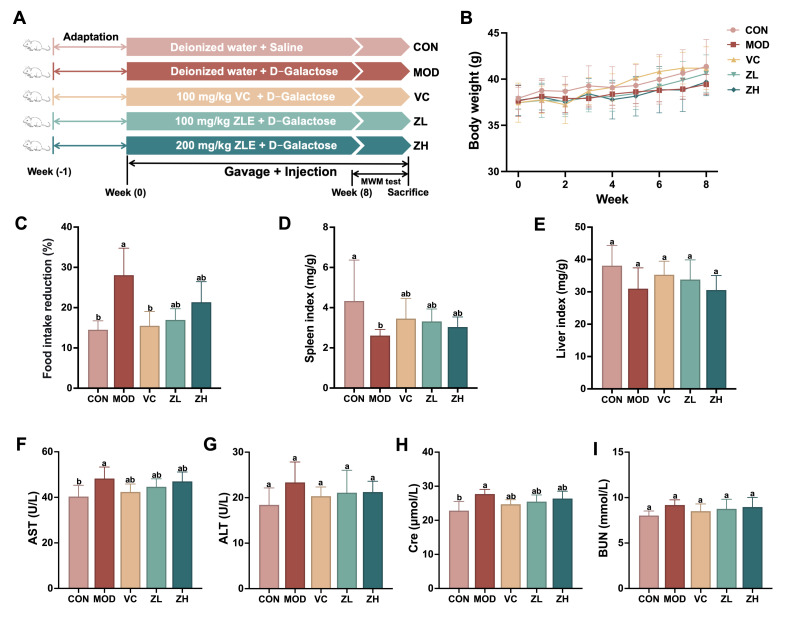
Effect of ZLE on the D-galactose-induced aging mice. (**A**) Animal experiment design. (**B**) Body weight. (**C**) Food intake reduction. (**D**) Spleen index. (**E**) Liver index. (**F**–**I**) The levels of AST, ALT, Cre, and BUN in serum. Different lowercase letters denote statistically significant differences (*p* < 0.05) between groups.

The animal degsin was shown in Figure 3A. The body weight of mice was monitored weekly for 8 weeks. There was no significant difference in the final body weight among all groups at the end of the experiment (Figure 3B). Nevertheless, the MOD group exhibited a slower body weight gain rate starting from the 4th week, while the ZL group maintained a relatively higher body weight compared with the MOD group. These results indicated that ZL intervention alleviated the delayed weight gain in D-galactose-induced aging mice to a certain extent. As shown in Figure 3C, the food intake was significantly reduced in the MOD group, whereas this abnormal change was ameliorated after ZLE intervention. The organ index, an important indicator for evaluating the physiological status of mice, indirectly reflects the growth and development of the body [28]. As shown in Figure 3D,E, the downward trend of spleen and liver indices in the MOD group was partially ameliorated by intervening with ZLE. In addition, the AST and Cre levels were significantly increased in the MOD group, suggesting that the D-galactose induced liver and kidney function impairment (Figure 3F–I) [29]. Importantly, ZLE intervention showed a downward trend in AST, ALT, Cre, and BUN levels, indicating that ZLE exhibited good biosafety for the liver and kidney [30]. Collectively, these results demonstrated that ZLE improved the physiological status, organ development, and key metabolic functions of D-galactose-induced aging mice.

### 3.7. ZLE Enhanced Spatial Learning and Memory Abilities in D-Galactose-Induced Aging Mice

**Figure 4:**
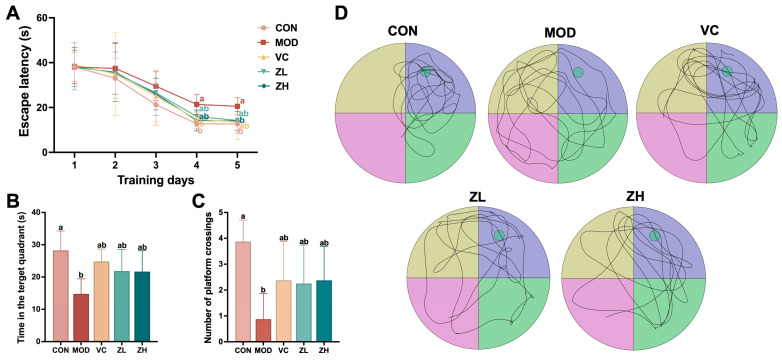
The Morris Water Maze (MWM) test. (**A**) Escape latency during training days, (**B**) time in the target quadrant, (**C**) number of platform crossings, (**D**) swimming trajectories. The circular pool was divided into four quadrants (colored differently for visual distinction), and the green dot marked the location of the hidden platform. Different lowercase letters denote statistically significant differences (*p* < 0.05) between groups.

The Morris Water Maze (MWM) test was performed to assess the spatial learning and memory abilities of mice [31]. As shown in Figure 4A, the escape latency gradually declined across training days in all groups, indicating a progressive improvement in spatial learning ability. During the first 3 days of training, there was no significant difference in escape latency among all groups. However, the ZH groups exhibited significantly shorter escape latency compared with the MOD group on day 5 (*p* = 0.0394). During the probe trial, the time spent in the target quadrant and the number of platform crossings were increased in the ZL and ZH groups (Figure 4B,C). As shown in Figure 4D, the swimming trajectories of the CON group were concentrated and mainly distributed in the target quadrant, demonstrating a clear platform-seeking strategy. In contrast, the disorganized and extensive exploratory patterns without directional preference for the target quadrant were exhibited in the MOD group. Notably, the swimming trajectories of the ZL and ZH groups were more focused on the target quadrant than those of the MOD group, suggesting a significant improvement in spatial learning and memory abilities. Collectively, these results demonstrated that D-galactose significantly impaired the spatial learning and memory abilities of mice, which were effectively ameliorated by ZLE intervention.

### 3.8. ZLE Ameliorated Hippocampal Neuronal Morphology in D-Galactose-Induced Aging Mice

**Figure 5:**
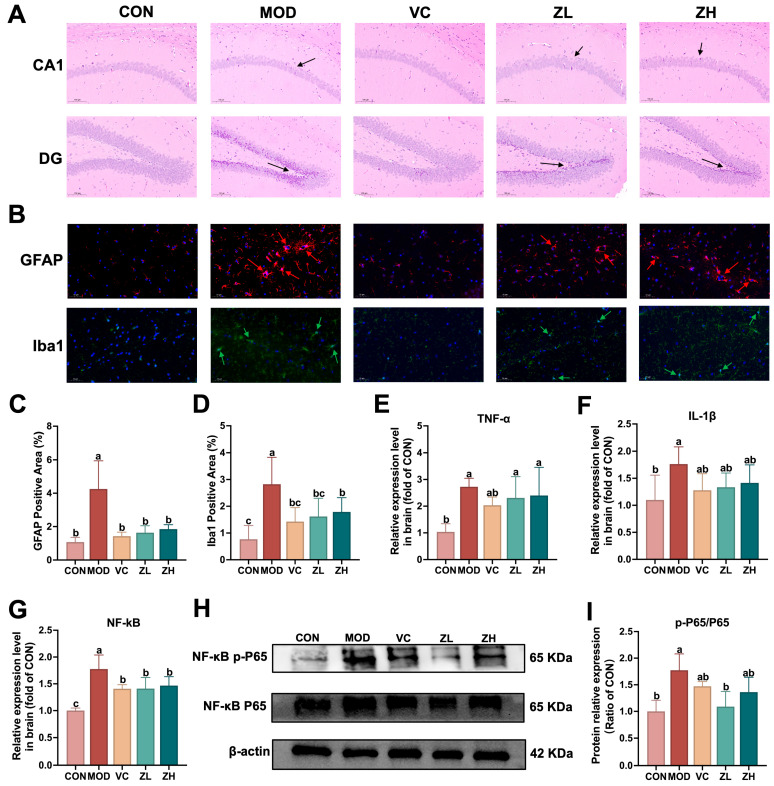
(**A**) Representative hematoxylin and eosin (H&E)-stained images of brain tissues. Upper panel: hippocampal CA1 region; lower panel: hippocampal DG region (scale bar = 100 μm). Arrows indicate representative pathological changes, including cytoplasmic hyperchromatism and nuclear condensation. (**B**) Representative immunofluorescence (IF) staining of brain tissues. Upper panel: GFAP (red) in the hippocampus; lower panel: Iba1 (green) in the hippocampus (scale bar = 50 μm). Arrows highlight representative activated GFAP-positive astrocytes and Iba1-positive microglia. (**C**,**D**) Quantification analysis of GFAP- and Iba1-positive area. (**E**–**G**) The relative mRNA expression level of IL-1β, TNF-α, and NF-κB in brain tissues. (**H**,**I**) Western blot analysis of NF-κB p-P65 and NF-κB P65. Different lowercase letters denote statistically significant differences (*p* < 0.05) between groups.

H&E staining was conducted to assess the neuronal morphology in the hippocampal CA1 and dentate gyrus (DG) regions. As shown in Figure 5A, neurons displayed a well-organized and compact arrangement with intact cellular morphology, indicating a healthy neuronal structure was observed in the CON group. In contrast, cytoplasmic hyperchromatism and nuclear condensation were exhibited in the MOD group, which was alleviated by ZLE intervention. In addition, the regulatory effects of the ZLE on neuroinflammation in D-galactose-induced aging mice were investigated by immunofluorescence (IF) staining of the brain tissues. Glial fibrillary acidic protein (GFAP, red fluorescence) was used to assess the activation status of astrocytes [32]. Ionized calcium-binding adapter molecule 1 (Iba1, green fluorescence), a calcium-binding protein specifically expressed in microglia, served as a specific marker for microglial cells [33]. Previous studies have shown that neuroinflammation is characterized by the increased area and abnormal activation of microglia and astrocytes [34]. Therefore, the IF staining of GFAP and Iba1 was conducted to elucidate the regulatory role of ZLE in neuroinflammation. As shown in Figure 5B–D, the area of GFAP- and Iba1-positive cells was significantly increased in the MOD group (*p* < 0.01), which demonstrated a significant activation of glial cells and an obvious neuroinflammatory response in the brain tissue. However, the areas of GFAP and Iba1 positive signals were markedly reduced following ZLE intervention compared with the MOD group (*p* < 0.01). These results suggested that ZLE effectively suppressed the excessive activation of astrocytes and microglia in the D-galactose-induced aging mice.

### 3.9. ZLE Improved Neuroinflammation in D-Galactose-Induced Aging Mice

As shown in Figure 5E,F, the relative expression levels of TNF-α and IL-1β in the brain tissues were significantly elevated in the MOD group. After intervention with ZLE, the relative mRNA expression levels of TNF-α and IL-1β exhibited a reduction without significant differences. These results suggested that ZLE partially ameliorated the neuroinflammation in the brain tissues of D-galactose-induced aging mice. Furthermore, the relative mRNA expression level of NF-κB in brain tissues was significantly increased in the MOD group, indicating that the NF-κB-mediated inflammatory signaling pathway was markedly activated (Figure 5G). In contrast, ZL and ZH treatment significantly reduced NF-κB transcription (*p* < 0.05), indicating that ZLE intervention inhibited the excessive activation of the NF-κB inflammatory signaling pathway. These findings were further verified at the protein level by Western blot. As shown in Figure 5H,I, the expression of p-P65 protein and the p-P65/P65 ratio were markedly higher than in the CON group (*p* = 0.021), indicating that D-galactose promoted P65 phosphorylation and thereby activated the NF-κB pathway. After low-dose ZLE intervention, the p-P65/P65 ratio was significantly reduced (*p* = 0.043), suggesting that the P65 phosphorylation was effectively inhibited. Collectively, these results demonstrated that ZLE suppressed the overactivation of the NF-κB pathway, downregulated the expression of the downstream inflammatory factors IL-1β and TNF-α, and ultimately alleviated the neuroinflammation in D-galactose-induced aging mice.

### 3.10. ZLE Attenuated the Oxidative Stress in D-Galactose-Induced Aging Mice

**Figure 6:**
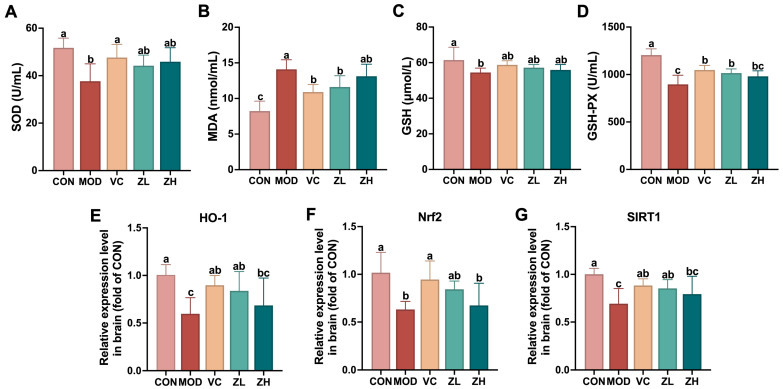
Effects of ZLE on the levels of SOD (**A**), MDA (**B**), GSH (**C**), and GSH-PX (**D**) in the serum. The relative mRNA expression level of HO-1 (**E**), Nrf2 (**F**) and SIRT1 (**G**) in brain tissues. Different lowercase letters denote statistically significant differences (*p* < 0.05) between groups.

As shown in Figure 6A–D, the levels of SOD, GSH, and GSH-PX in serum were significantly decreased in D-galactose-induced mice (*p* < 0.05), while the MDA content was markedly increased (*p* < 0.01). These findings indicated that the model mice exhibited obvious oxidative stress injury. Notably, the MDA and GSH-PX levels were significantly restored in the ZL group (*p* < 0.05). Collectively, these results suggested that ZLE effectively ameliorated oxidative stress injury in D-galactose-induced aging mice. To further investigate the effects of ZLE on key antioxidant-related signaling pathways, the expression levels of SIRT1, Nrf2, and HO-1 in brain tissue were examined. The results showed that the relative mRNA expression levels of HO-1 and SIRT1 were significantly upregulated in the ZL group, and the downregulation of Nrf2 expression was alleviated, demonstrating that the ZL group effectively activated the antioxidant signaling pathway in the brain tissues of D-galactose-induced mice.

## 4. Discussion

Age-related physiological decline, particularly cognitive impairment and neuronal damage driven by oxidative stress and neuroinflammation, has prompted extensive research on natural dietary components with potential anti-aging properties [35]. This study systematically investigated the multifunctional bioactivities of ZLE, including in vitro neuroprotective effects on HT-22 neuronal cells and in vivo regulatory effects on aging-related pathological alterations. These findings provided a scientific foundation for subsequent mechanistic exploration of ZLE and evaluation of its potential activities.

Oxidative stress is a pathophysiological state resulting from an imbalance between ROS production and antioxidant defense systems [36]. The core characteristic involves the excessive accumulation of ROS, which triggers lipid peroxidation, protein denaturation, and DNA damage, ultimately leading to cellular dysfunction or even apoptosis [37]. Enzyme-mediated antioxidant activity constitutes the central defense mechanism of the antioxidant system. SOD and CAT, as key enzymes in this system, maintain cellular ROS homeostasis through synergistic action [38]. Here, ZLE effectively scavenged superoxide anions, DPPH, and ABTS^+^ radicals at high concentrations (5 mg/mL), supporting its subsequent cytoprotective effects. Importantly, the antioxidant activity of ZLE contributed to cellular protection in H_2_O_2_-induced HT-22 cells: ZLE intervention enhanced the viability of H_2_O_2_-induced HT-22 cells. This protective effect was associated with the ability of ZLE to reduce intracellular ROS accumulation and upregulate the mRNA expression levels of SOD and CAT, thereby strengthening the endogenous antioxidant defense system of neuronal cells. The dual actions of directly scavenging free radicals and enhancing endogenous antioxidant enzyme expression suggested that ZLE had potential for alleviating oxidative stress-induced neuronal damage. Additionally, oxidative stress is frequently accompanied by inflammation, and these two processes interact to form a vicious cycle, thereby further exacerbating cellular damage [39]. Consistent with this, ZLE alleviated H_2_O_2_-triggered neuroinflammation in HT-22 cells by suppressing the mRNA expression of pro-inflammatory cytokines. These results demonstrated that ZLE exerted the dual protective effects against oxidative and inflammatory injury in neuronal cells, supporting its potential neuroprotective properties and providing experimental evidence for subsequent in vivo efficacy evaluation.

Based on the above in vitro research findings, the physiological regulation and underlying mechanisms of ZLE were investigated by establishing a D-galactose-induced aging mouse model. Organ index is a core quantitative indicator reflecting organ morphological integrity and physiological homeostasis [28]. Studies have shown that persistent oxidative stress induces functional degeneration and structural damage in multiple organs [40]. The liver, a central organ for metabolism, detoxification, antioxidant defense, and synthesis, is closely associated with oxidative stress-related damage [41]. The spleen, as the primary peripheral immune organ, plays a crucial role in immune cell proliferation, differentiation, and immune response regulation, making it a key biomarker for evaluating systemic immune function. The results indicated that ZLE improved the liver and spleen indices and alleviated the reduction in body weight gain and food intake in D-galactose-induced aging mice. These beneficial effects were likely attributed to the ability of ZLE to regulate systemic oxidative stress [42]. In the model mice, serum antioxidant enzyme activities, including SOD, GSH, and GSH-PX, were reduced, while serum MDA content was increased. Notably, these alterations were improved by ZLE administration, helping to restore antioxidant balance and mitigate oxidative damage. Moreover, the systemic antioxidant effects of ZLE were further explored at the molecular level in brain tissue. Specifically, ZLE upregulated the expression of the SIRT1-Nrf2-HO-1 signaling axis. Extensive studies have demonstrated that the dysregulation of SIRT1, Nrf2, and HO-1 pathways is closely associated with neurological dysfunction, cognitive impairment, and neuronal death. As a key regulator of oxidative stress, SIRT1 deacetylates Nrf2, promoting its nuclear translocation and thereby activating HO-1 transcription [43]. The SIRT1/Nrf2/HO-1 pathway contributed to enhancing the intrinsic antioxidant capacity of the brain, which may protect against oxidative damage and reduce neuronal loss, potentially improving age-related neurological disorders.

Subsequently, the research focused on exploring the potential mechanisms underlying the protective effects of ZLE in the CNS. The hippocampus, as a key brain region responsible for spatial learning and memory, is highly sensitive to oxidative stress and neuroinflammation [44]. Here, ZLE intervention ameliorated the hippocampal neuronal degeneration, protected the morphological integrity of DG neurons, and significantly reduced the overactivation of GFAP and Iba1 positive cells in the D-galactose-induced aging mice, which was consistent with previous findings [45,46,47]. Meanwhile, the pro-inflammatory cytokine expression in the brain was downregulated by ZLE treatment, indicating effective suppression of neuroinflammation. Similarly, Hu et al. found that natural flavonoid abrogated the excessive activation of astrocytes and microglia, thereby attenuating neuroinflammation to protect neurons in mice with cognitive deficits [48]. These results demonstrated that ZLE possessed a neuroprotective effect to some extent by reducing central nervous inflammation and maintaining the structural integrity of hippocampal neurons.

Mechanistically, the NF-κB signaling pathway acts as a key regulator of neuroinflammation. Many studies have demonstrated that the persistent activation of the NF-κB drives the transcription and release of pro-inflammatory cytokines such as TNF-α and IL-1β, thereby inducing glial cell activation and neuronal apoptosis, constituting a critical molecular mechanism underlying age-related neuroinflammation [49]. Our study found that ZLE significantly downregulated the phosphorylation level of NF-κB p65 (pp65) and the mRNA expression of NF-κB in brain tissue (*p* < 0.05), as well as reduced the level of pro-inflammatory cytokine IL-1β and TNF-α, which indicated the inhibitory effect of ZLE on NF-κB signaling activation. Similarly, Wang et al. reported that phenolic compounds from *Vaccinium* species exerted anti-neuroinflammatory effects by inhibiting the NF-κB pathway [50]. Furthermore, the neural structural protection and neuroinflammation suppression were associated with the improvements in cognitive function. In the MWM test, mice in the ZLE groups exhibited significant behavioral improvements compared with the MOD group, including reduced escape latency, prolonged target quadrant residence time, increased the number of platform crossings, and more targeted swimming trajectories. These behavioral findings were consistent with previous studies, in which nfkb1^−^/^−^ mice exhibited spatial memory deficits, supporting the contribution of NF-κB-mediated neuroinflammation to age-related spatial learning and memory impairments [51]. Thus, the neuroprotective effects of ZLE were not only observed at the cellular and molecular levels but also translated into physiological functional improvements, reflecting its protective efficacy in the D-galactose-induced mouse model from molecular mechanisms to behavioral phenotypes. In conclusion, ZLE intervention protected neuronal structural integrity and improved spatial learning and memory deficits, which might be attributed to the inhibition of the NF-κB signaling pathway. These regulatory effects contribute to the neuroprotective and anti-aging-related activities of ZLE in aging mice model to some extent.

The above protective activities of ZLE might be attributed to its unique phytochemical composition. Notably, ZLE was rich in iridoid glycosides, organic acids, flavonoids, and other bioactive constituents. Previously, Afifi et al. have reported that iridoids-rich fraction exhibited potential neuroprotective activity and alleviated neurodegeneration in mice [52]. Numerous studies have demonstrated that flavonoids exerted potential effects against neurological disorders, mainly by suppressing oxidative stress, preserving cellular structure and function, and regulating inflammatory responses [53,54]. Therefore, we suggested that these chemical characteristics might constitute the material basis for its neuroprotective and anti-aging effects.

However, this study exhibited several limitations that warrant further exploration and improvement. First, the bioavailability and brain distribution of ZLE remain unclear. The ability of ZLE to cross the blood–brain barrier and accumulate in functional brain regions remains to be determined, which is critical for understanding its central mechanism of action. Second, the present study used a fixed dosage of ZLE, the optimal dose–response relationship has not been fully established. Further research is needed to determine the effective and safe dose range for long-term intervention. Third, cellular experiments were performed without positive control. Incorporating the classic reference compounds such as vitamin C, N-Acetylcysteine, or Trolox in future studies will facilitate a more comprehensive assessment of biological activities of ZLE. In addition, the contributions of the base liquor and plant extracts to the bioactivity of ZLE have not been distinguished, and inclusion of a liquor-only control group in future studies will help clarify the specific source of the effects. These limitations highlight important directions for further research to better characterize the active components, pharmacokinetic properties, and clinical translational potential of ZLE as a functional food or nutritional intervention.

## 5. Conclusions

This study demonstrated that Zhuyeqing liquor extract (ZLE) exhibited antioxidant, anti-neuroinflammatory, and neuroprotective effects in vitro and in vivo. The results showed that ZLE protected HT-22 cells against H_2_O_2_-induced oxidative stress and cell cycle arrest by reducing ROS accumulation, upregulating antioxidant enzyme expression and inhibiting pro-inflammatory cytokine release. Meanwhile, ZLE ameliorated the aging-related physiological abnormalities in D-galactose-induced mice, including enhancing organ indices, preserving hippocampal neuronal morphology, suppressing the excessive activation of astrocytes and microglia, and improving spatial learning and memory capacity. Mechanistically, ZLE alleviated the aging-related oxidative stress and neuroinflammation by activating the SIRT1-Nrf2-HO-1 signaling pathway and inhibiting the NF-κB pathway. These findings suggest the potential of ZLE as a natural functional dietary ingredient in alleviating age-related oxidative stress and neuroinflammation and provide a scientific foundation for developing traditional liquor extracts into modern functional foods.

## Data Availability

The original contributions presented in the study are included in the article/Supplementary Materials; further inquiries can be directed to the corresponding authors. Additionally, since the ZLE used in this study is the same batch as in our previous related work (not yet published), the chemical characterization data are consistent with the previous paper. The Supplementary Materials are intended solely to illustrate the sample composition and do not present any novel findings.

## Supplementary Materials

The following supporting information can be downloaded at: https://www.mdpi.com/article/10.3390/foods15122085/s1, Figure S1: Total ion chromatogram (TIC) of ZLE. (A) ESI^+^ mode; (B) ESI^−^ mode; Table S1: Phytochemical profile of ZLE characterized by UPLC-Q-Exactive HF-MS in positive ion mode (ESI^+^); Table S2: Phytochemical profile of ZLE characterized by UPLC-Q-Exactive HF-MS in negative ion mode (ESI^−^).

## Appendix A

**Table A1:** The gene-specific primers for RT-qPCR analysis.

Genes	Forward (5′-3′)	Reverse (5′-3′)
*β-actin*	GGCTGTATTCCCCTCCATCG	CCAGTTGGTAACAATGCCATGT
*TNF-α*	GTCCTAGTAACTGGCACTTC	GAGCAGAGTGATTCGTAGAG
*IL-1β*	GAAGAAGAGCCCATCCTCTG	GTTCATCTCGGAGCCTGTAG
*IL-6*	ACTTCCATCCAGTTGCCTTCTTG	TGTTGGGAGTGGTATCCTCTGTG
*CAT*	CCTATTGCCGTTCGATTCTC	TCCCACAAGATCCCAGTTAC
*SOD*	TACAACTCAGGTCGCTCTTC	CAAAGTCACGCTTGATAGCC
*NF-κB*	GGAGACATCCTTCCGCAAAC	AGGTCCTTCCTGCCCATAAC
*SIRT1*	CACATGCCAGAGTCCAAGTTTAGAAG	AATCCAGATCCTCCAGCACATTCG
*Nrf2*	GTTGCCACCGCCAGGACTAC	CTTGTACCGCCTCGTCTGGAC
*HO-1*	ACCGCCTTCCTGCTCAACATTG	CTGTGAGGGACTCTGGTCTTTGTG

Notes: All primers were synthesized by Tsingke Biotechnology Co., Ltd., Beijing, China.
