# Supplementary material for: Zhuyeqing Liquor Extract Ameliorates Oxidative Stress and Neuroinflammation in D-Galactose-Induced Aging Mice Model
Source: Foods. 2026 Jun 9;15(12):2085. doi: 10.3390/foods15122085 (PMC13298492; doi:10.3390/foods15122085)
Supplement: Supplementary file 1 [file foods-15-02085-s001.zip › foods-4323565-supplementary.pdf]

foods

MDPI

Supporting Information

Zhuyeqing liquor extract ameliorates oxidative stress and neuroinflammation in D-galactose-induced aging mice model

Ying Han [1#], Lin Wang [2#], Pan Zhen [1], Xiaoxiao Li [1], Rong Liu [1], Hanyue Fu [2], Xiang Li [2], Bingye Xu [2], Fan Wei [2],Bowei Zhang [2,*], Shuo Wang [2,*]

[1] Shanxi XinghuaCun Fen Jiu Distillery Co., Ltd, Shanxi 033000, China
[2] Tianjin Key Laboratory of Food Science and Health, School of Medicine, Nankai University, Tianjin 300071, China

Note: Since the ZLE used in this study is the same batch as in our previous related work (not yet published), the chemical characterization data are consistent with the previous paper. This supplementary material is intended solely to illustrate the sample composition and does not present any novel findings.

Figure S1. Total ion chromatogram (TIC) of ZLE. (A) ESI⁺ mode; (B) ESI⁻ mode.

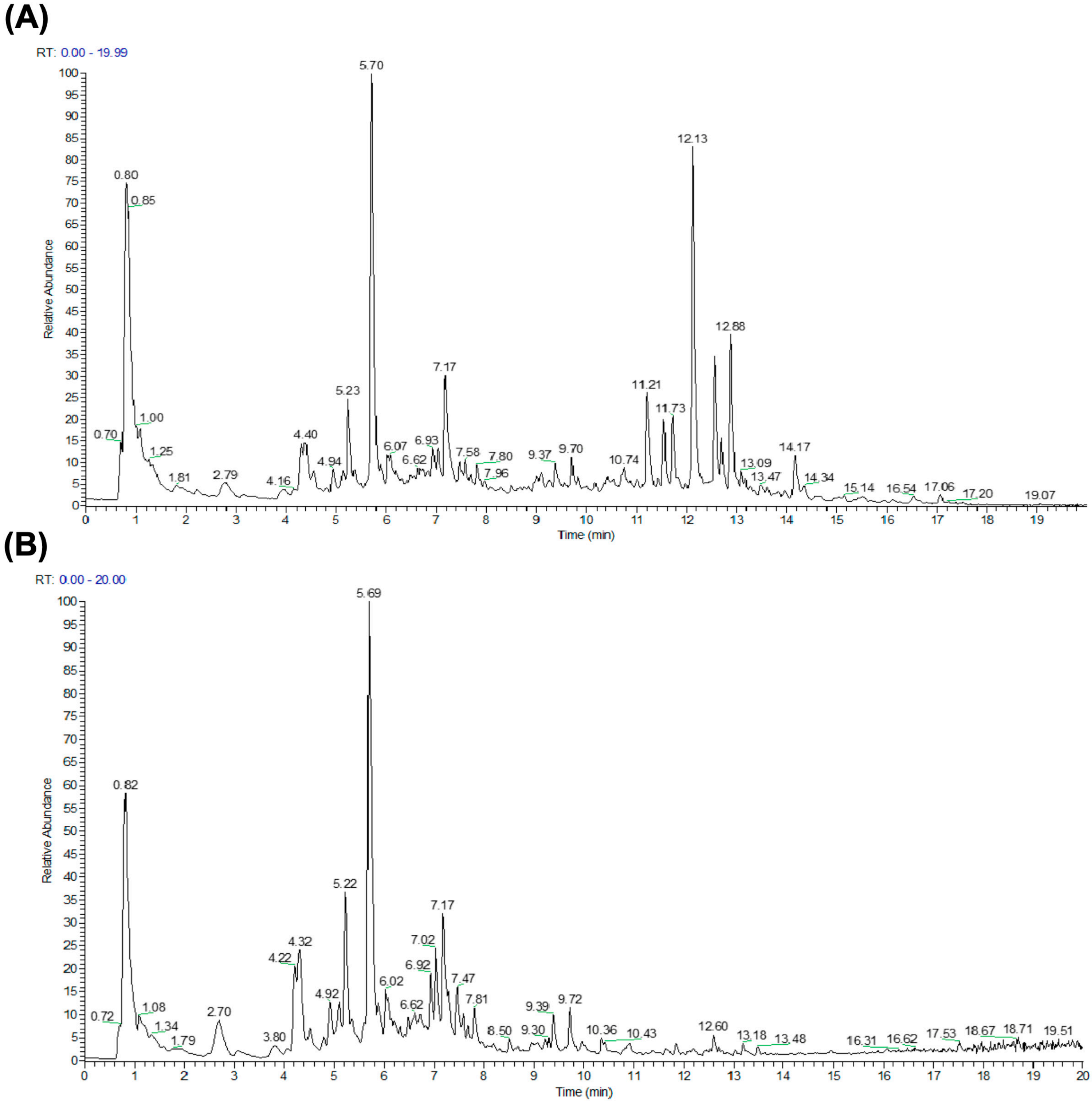

Table S1. Phytochemical profile of ZLE characterized by UPLC-Q-Exactive HF-MS in positive ion mode (ESI$^+$).

Name	Formula	CAS_num	Class	Annot. DeltaMass (ppm)	RT (min)	Area	Concentration (μ g/mL)	Relative Contents (%)
4-Methoxycinnamic acid	C10 H10 O3	943-89-5	Cinnamic acids and derivatives	1.55	12.176	6689764934	77.703	9.879%
Ethyl 4-methoxycinnamate	C12 H14 O3	24393-56-4	Cinnamic acids and derivatives	1.59	12.176	4935855733	57.331	7.289%
4-Methylumbelliferone	C10 H8 O3	90-33-5	Coumarins and derivatives	1.53	5.754	4523631752	52.543	6.680%
DL-Stachydrine	C7 H13 N O2	4136-37-2	Carboxylic acids and derivatives	1.65	0.883	3826094635	44.441	5.650%
Nobiletin	C21 H22 O8	478-01-3	Flavonoids	1.12	11.253	3442880069	39.990	5.084%
Cinnamic acid	C9 H8 O2	140-10-3	Cinnamic acids and derivatives	1.72	5.754	2777244236	32.258	4.101%
p-Cresol	C7 H8 O	106-44-5	Phenols	2.11	1.114	2581256986	29.982	3.812%
Tangeritin	C20 H20 O7	481-53-8	Flavonoids	0.9	11.776	2448156035	28.436	3.615%
Choline	C5 H13 N O	62-49-7	Organonitrogen compounds	4.94	0.819	2043393227	23.735	3.018%
{[{[(2R,3S,4R)-5-(4-Amino-2-oxo-1(2H)-pyrimidinyl)-3,4-dihydroxytetrahydro-2-furanyl]methoxy}(hydroxy)phosphoryl]amino}acetic acid	C11 H17 N4 O9 P	NA	Organooxygen compounds	-2.37	0.836	1840434618	21.377	2.718%
Acetophenone	C8 H8 O	98-86-2	Organooxygen compounds	3.09	5.754	1727674973	20.067	2.551%
D-(+)-Proline	C5 H9 N O2	4305-67-3	Carboxylic acids and derivatives	3.53	0.86	1721794218	19.999	2.543%
Cinnamaldehyde	C9 H8 O	104-55-2	Cinnamaldehydes	2.18	12.177	1631689691	18.952	2.410%
1,4-Naphthoquinone	C10 H6 O2	130-15-4	Naphthalenes	1.52	5.755	1299284652	15.092	1.919%
DL-Arginine	C6 H14 N4 O2	7200-25-1	Carboxylic acids and derivatives	1.43	0.84	1296845993	15.063	1.915%
Bis(2,2-dihydroxyethyl) hydrogen phosphate	C4 H11 O8 P	NA	Organic phosphoric acids and derivatives	1.74	0.822	1230886102	14.297	1.818%
Isoalantolactone	C15 H20 O2	470-17-7	Prenol lipids	1.27	12.749	976065449	11.337	1.441%
Methocarbamol	C11 H15 N O5	532-03-6	Phenol ethers	1.75	1.872	922060605.3	10.710	1.362%
7-Methoxycoumarin	C10 H8 O3	531-59-9	Coumarins and derivatives	1.6	5.286	901536409	10.472	1.331%
Jasminoside B	C16 H26 O8	214125-04-9	Organooxygen compounds	0.99	4.445	819623622.4	9.520	1.210%
Sarracenin	C11 H14 O5	59653-37-1	Dioxanes	0.88	5.754	801599934.5	9.311	1.184%
Cinnamyl alcohol	C9 H10 O	104-54-1	Cinnamyl alcohols	2.3	4.445	791592719.7	9.195	1.169%
(-)-Caryophyllene oxide	C15 H24 O	1139-30-6	Prenol lipids	1.09	11.277	752657910.6	8.742	1.112%
Hesperidin	C28 H34 O15	520-26-3	Flavonoids	1.3	7.226	643838823.6	7.478	0.951%
Lawsone	C10 H6 O3	83-72-7	Naphthalenes	2	7.864	617932379.1	7.177	0.913%
Coumarin	C9 H6 O2	91-64-5	Coumarins and derivatives	1.4	7.234	593609141	6.895	0.877%
Chlorogenic acid	C16 H18 O9	202650-88-2	Organooxygen compounds	0.83	5.179	567259010	6.589	0.838%
Entecavir	C12 H15 N5 O3	142217-69-4	Nucleoside and nucleotide analogues	-3.98	0.876	506158441.7	5.879	0.747%
Adenine	C5 H5 N5	73-24-5	Imidazopyrimidines	1.78	0.905	488288494	5.672	0.721%
Chamazulene	C14 H16	529-05-5	Prenol lipids	1.99	12.933	477529040.6	5.547	0.705%
OZ4000000	C16 H26 O7	109-17-1	Carboxylic acids and derivatives	0.87	6.13	459599094.5	5.338	0.679%
Lyoniside	C27 H36 O12	34425-25-7	Lignan glycosides	1.68	7.639	456634978.1	5.304	0.674%
Betaine	C5 H11 N O2	107-43-7	Carboxylic acids and derivatives	2.96	0.847	440244380.9	5.114	0.650%
D-(+)-Pipecolinic acid	C6 H11 N O2	1723-00-8	Carboxylic acids and derivatives	1.96	0.903	437051138.5	5.076	0.645%
Genistin	C21 H20 O10	529-59-9	Isoflavonoids	1.3	7.257	393713932	4.573	0.581%

5-O-Demethylnobiletin	C20 H20 O8	2174-59-6	Flavonoids	0.73	12.253	348335394.3	4.046	0.514%
Oleamide	C18 H35 N O	301-02-0	Fatty Acyls	0.56	17.114	340830172.9	3.959	0.503%
L-Norleucine	C6 H13 N O2	327-56-0	Carboxylic acids and derivatives	2.45	1.4	327704703.1	3.806	0.484%
7-Hydroxycoumarine	C9 H6 O3	93-35-6	Coumarins and derivatives	1.47	5.285	301933033.6	3.507	0.446%
MFCD00059002	C16 H35 N O2	1541-67-9	Organonitrogen compounds	0.97	10.512	283230669.7	3.290	0.418%
Crocetin	C20 H24 O4	27876-94-4	Prenol lipids	1.02	7.077	259139457.6	3.010	0.383%
α-Eleostearic acid	C18 H30 O2	544-73-0	Fatty Acyls	0.97	13.23	258548797	3.003	0.382%
Glycitin	C22 H22 O10	40246-10-4	Isoflavonoids	0.9	8.996	250906324.8	2.914	0.371%
L-Phenylalanine	C9 H11 N O2	150-30-1	Carboxylic acids and derivatives	2.05	2.277	237769681.7	2.762	0.351%
Cynaroside	C21 H20 O11	5373-11-5	Flavonoids	0.99	6.788	225806760.1	2.623	0.333%
Apigenin	C15 H10 O5	520-36-5	Flavonoids	0.67	7.226	222074053.7	2.579	0.328%
Sinapinic acid	C11 H12 O5	530-59-6	Cinnamic acids and derivatives	1.56	5.718	210360950.2	2.443	0.311%
Wilforlide A	C30 H46 O3	84104-71-2	Prenol lipids	0.96	9.757	207033026.5	2.405	0.306%
(-)-Camphor	C10 H16 O	464-48-2	Prenol lipids	1.35	11.575	193266553.7	2.245	0.285%
Rutin	C27 H30 O16	153-18-4	Flavonoids	2.05	6.537	183600908.8	2.133	0.271%
7,8-Dihydroxy-4-methylcoumarin	C10 H8 O4	2107-77-9	Coumarins and derivatives	1.75	3.993	182055007.5	2.115	0.269%
5,7-Dihydroxy-2-(4-hydroxyphenyl)-6,8-bis[3,4,5-trihydroxy-6-(hydroxymethyl)tetrahydro-2H-pyran-2-yl]-4H-chromen-4-one	C27 H30 O15	23666-13-9	Flavonoids	1.2	5.737	171525859.3	1.992	0.253%
Artemetin	C20 H20 O8	479-90-3	Flavonoids	0.97	12.109	168068694.9	1.952	0.248%
Methyl isonicotinate	C7 H7 N O2	2459-09-8	Pyridines and derivatives	1.39	0.856	159175438.8	1.849	0.235%
lactide	C6 H8 O4	95-96-5	Dioxanes	2.36	0.87	156740730.8	1.821	0.231%
13(S)-HOTrE	C18 H30 O3	87984-82-5	Fatty Acyls	1.29	13.513	146248124.3	1.699	0.216%
(3β,5ξ,9ξ)-3,6,19-Trihydroxyurs-12-en-28-oic acid	C30 H48 O5	NA	Prenol lipids	1.34	11.887	141608233	1.645	0.209%
Isoferulic acid	C10 H10 O4	25522-33-2	Cinnamic acids and derivatives	1.63	2.826	136639414.3	1.587	0.202%
2-Amino-1,3,4-octadecanetriol	C18 H39 N O3	554-62-1	Organonitrogen compounds	1.1	10.56	136576856.3	1.586	0.202%
Naringenin	C15 H12 O5	480-41-1	Flavonoids	0.67	6.98	136570066.8	1.586	0.202%
Hexadecanamide	C16 H33 N O	629-54-9	Fatty Acyls	0.93	16.597	133472793.5	1.550	0.197%
Linolenic acid ethyl ester	C20 H34 O2	1191-41-9	Fatty Acyls	0.8	16.554	132237725.9	1.536	0.195%
Tectoridin	C22 H22 O11	611-40-5	Isoflavonoids	1.21	7.441	121844895.8	1.415	0.180%
Vitexin	C21 H20 O10	3681-93-4	Flavonoids	1.53	6.597	118077848.4	1.372	0.174%
L-Pyroglutamic acid	C5 H7 N O3	98-79-3	Carboxylic acids and derivatives	2.68	1.063	115533647.2	1.342	0.171%
Kaempferol	C15 H10 O6	520-18-3	Flavonoids	0.79	9.021	115161245.1	1.338	0.170%
4,5-Dicaffeoylquinic acid	C25 H24 O12	14534-61-3	Organooxygen compounds	1.4	7.206	114769234.1	1.333	0.169%
Dehydrodiisoeugenol	C20 H22 O4	2680-81-1	2-arylbenzofuran flavonoids	1.16	13.327	112188519.6	1.303	0.166%
Ethyl caffeate	C11 H12 O4	102-37-4	Cinnamic acids and derivatives	1.15	9.439	107137897.8	1.244	0.158%
Didymin	C28 H34 O14	14259-47-3	Flavonoids	1.52	8.557	105528359.7	1.226	0.156%
L-Tyrosine	C9 H11 N O3	556-03-6	Carboxylic acids and derivatives	1.97	1.307	105496358.1	1.225	0.156%
(1ξ)-1,5-Anhydro-1-[2-(3,4-dihydroxyphenyl)-5,7-dihydroxy-4-oxo-4H-chromen-8-yl]-D-galactitol	C21 H20 O11	NA	Flavonoids	1.3	6.165	104101349.8	1.209	0.154%
Quercetin-3β-D-glucoside	C21 H20 O12	482-35-9	Flavonoids	1.48	6.773	103665937	1.204	0.153%

Linoleoyl Ethanolamide	C20 H37 N O2	68171-52-8	Organonitrogen compounds	1.2	14.619	103030992.7	1.197	0.152%
4-Coumaric acid	C9 H8 O3	7400-08-0	Cinnamic acids and derivatives	1.49	10.412	101351757.9	1.177	0.150%
Methyl cinnamate	C10 H10 O2	103-26-4	Cinnamic acids and derivatives	1.17	5.754	96799281.9	1.124	0.143%
9-Oxo-10(E),12(E)-octadecadienoic acid	C18 H30 O3	54232-59-6	Fatty Acyls	1.16	13.666	92492450.25	1.074	0.137%
Bis(4-ethylbenzylidene)sorbitol	C24 H30 O6	79072-96-1	Dioxanes	0.87	12.027	92027026.37	1.069	0.136%
2-AMINO-3-(2-CHLORO-PHENYL)-PROPIONIC ACID	C9 H10 Cl N O2	14091-11-3	The internal standard	1.79	4.572	172187444.9	1.000	0.000%
Quercetin	C15 H10 O7	117-39-5	Flavonoids	1.48	6.535	85966990.98	0.999	0.127%
3-Butylidenephthalide	C12 H12 O2	72917-31-8	Isocoumarans	2.31	12.634	82410203.6	0.957	0.122%
Isophorone	C9 H14 O	78-59-1	Organooxygen compounds	1.69	9.879	81300820.08	0.944	0.120%
Glycitein	C16 H12 O5	40957-83-3	Isoflavonoids	0.47	11.283	78409248.42	0.911	0.116%
Furaltadone	C13 H16 N4 O6	139-91-3	Furans	-2.69	0.869	74201816.48	0.862	0.110%
N-(2,4-Dimethylphenyl)formamide	C9 H11 N O	60397-77-5	Benzene and substituted derivatives	1.65	4.469	72396300.18	0.841	0.107%
4-Methylumbelliferyl-α-D-glucopyranoside	C16 H18 O8	6160-78-7	Coumarins and derivatives	1.02	7.322	66841122.34	0.776	0.099%
2-Naphthylamine	C10 H9 N	91-59-8	Naphthalenes	2.17	5.509	62838158.54	0.730	0.093%
Ethyl ferulate	C12 H14 O4	4046-02-0	Cinnamic acids and derivatives	1.36	10.603	62083151.53	0.721	0.092%
Glabrolide	C30 H44 O4	10401-33-9	Prenol lipids	1.56	10.921	61863213.35	0.719	0.091%
L-Aspartic acid β-benzyl ester	C11 H13 N O4	2177-63-1	Carboxylic acids and derivatives	1.17	2.737	61176810.17	0.711	0.090%
9S,13R-12-Oxophytodienoic acid	C18 H28 O3	67204-66-4	Fatty Acyls	0.8	9.659	59918847.27	0.696	0.088%
Quercetin 3-O-β-D-Glucuronide	C21 H18 O13	22688-79-5	Flavonoids	1.42	6.761	59744703.61	0.694	0.088%
Ethyl benzoate	C9 H10 O2	93-89-0	Benzene and substituted derivatives	1.74	5.753	57497087.76	0.668	0.085%
Stearamide	C18 H37 N O	124-26-5	Carboximidic acids and derivatives	1.06	19.116	55895345.49	0.649	0.083%
2,3,4,9-Tetrahydro-1H-β-carboline-3-carboxylic acid	C12 H12 N2 O2	42438-90-4	Indoles and derivatives	1.91	5.51	55871645.89	0.649	0.083%
Scopoletin	C10 H8 O4	92-61-5	Coumarins and derivatives	2.44	5.421	55604044.43	0.646	0.082%
5,2'-Dihydroxy-6,7,8,6'-tetramethoxyflavone	C19 H18 O8	55084-08-7	Flavonoids	0.64	11.367	55437410.93	0.644	0.082%
2,4-Dimethylbenzaldehyde	C9 H10 O	15764-16-6	Benzene and substituted derivatives	2.18	5.928	54943880.81	0.638	0.081%
Nootkatone	C15 H22 O	4674-50-4	Prenol lipids	1.19	11.235	54081031.67	0.628	0.080%
Pantothenate	C9 H17 N O5	79-83-4	Organooxygen compounds	1.33	2.827	53415493.7	0.620	0.079%
Scutellarin	C21 H18 O12	27740-01-8	Flavonoids	0.93	6.81	50407858.71	0.585	0.074%
Prolylleucine	C11 H20 N2 O3	52899-07-7	Carboxylic acids and derivatives	1.71	1.156	50208731.63	0.583	0.074%
Gardenin B	C19 H18 O7	2798-20-1	Flavonoids	0.26	12.666	48310324.19	0.561	0.071%
Linarin	C28 H32 O14	480-36-4	Flavonoids	1.53	8.256	46232836.54	0.537	0.068%
Rhoifolin	C27 H30 O14	17306-46-6	Flavonoids	2.01	6.966	46139885.45	0.536	0.068%
Guanine	C5 H5 N5 O	73-40-5	Imidazopyrimidines	2.64	1.065	45988329.59	0.534	0.068%
Uracil	C4 H4 N2 O2	66-22-8	Diazines	4.22	1.26	43309615.18	0.503	0.064%
Limonin	C26 H30 O8	1180-71-8	Prenol lipids	1.26	10.971	42979607.84	0.499	0.063%
Guanosine	C10 H13 N5 O5	118-00-3	Purine nucleosides	0.87	1.349	42857037.75	0.498	0.063%

Vanillin	C8 H8 O3	121-33-5	Phenols	1.81	6.686	42804819.97	0.497	0.063%
1,2,3,4-Tetramethyl-1,3-cyclopentadiene	C9 H14	4249-10-9	Unsaturated hydrocarbons	2.89	4.44	42663267.65	0.496	0.063%
7-Methoxy-4-methylcoumarin	C11 H10 O3	2555-28-4	Coumarins and derivatives	1.48	11.496	41953170.43	0.487	0.062%
Nicotinamide	C6 H6 N2 O	98-92-0	Pyridines and derivatives	2.96	1.086	41214925.89	0.479	0.061%
Curcumol	C15 H24 O2	4871-97-0	Prenol lipids	0.97	13.012	40955613.08	0.476	0.060%
(1α,2α,3β,5ξ,9ξ,18ξ)-1,2,3,19-Tetrahydroxyurs-12-en-28-oic acid	C30 H48 O6	NA	Prenol lipids	1.28	12.251	40353933.26	0.469	0.060%
3',4'-Dihydroxyphenylacetone	C9 H10 O3	2503-44-8	Benzene and substituted derivatives	2.01	2.041	39719568.58	0.461	0.059%
Keracyanin	C27 H30 O15	18719-76-1	Flavonoids	2.29	6.553	38991706.38	0.453	0.058%
Corymboside	C26 H28 O14	73543-87-0	Flavonoids	1.71	6.078	38359608.96	0.446	0.057%
Isokaempferide	C16 H12 O6	1592-70-7	Flavonoids	0.87	9.974	38330602.4	0.445	0.057%
1-Linoleoyl glycerol	C21 H38 O4	2277-28-3	Fatty Acyls	0.9	13.305	38277281.73	0.445	0.057%
4-Ethynylaniline	C8 H7 N	14235-81-5	Benzene and substituted derivatives	3.62	4.576	38177880.23	0.443	0.056%
Ferulaldehyde	C10 H10 O3	20649-42-7	Phenols	1.65	7.764	37884372.18	0.440	0.056%
Sinapyl aldehyde	C11 H12 O4	4206-58-0	Phenols	1.57	2.574	37844433.62	0.440	0.056%
Ethyl nicotinate	C8 H9 N O2	614-18-6	Pyridines and derivatives	2	6.684	37223582.73	0.432	0.055%
Doxazosin	C23 H25 N5 O5	74191-85-8	Diazinanes	-1.7	6.43	36461111.25	0.424	0.054%
Nardosinone	C15 H22 O3	23720-80-1	Prenol lipids	1.27	6.677	33917794.95	0.394	0.050%
BMK methyl glycidate	C11 H12 O3	80532-66-7	Epoxides	1.97	5.206	32472297.41	0.377	0.048%
Kuromanin	C21 H20 O11	7084-24-4	Flavonoids	0.95	7.141	31976834.52	0.371	0.047%
Fraxetin	C10 H8 O5	574-84-5	Coumarins and derivatives	1.57	3.436	31741203.83	0.369	0.047%
trans-Anethole	C10 H12 O	4180-23-8	Phenol ethers	1.6	5.826	31464972.64	0.365	0.046%
4-Methoxybenzaldehyde	C8 H8 O2	123-11-5	Benzene and substituted derivatives	1.58	9.158	31454797.97	0.365	0.046%
(+/-)12(13)-DiHOME	C18 H34 O4	263399-35-5	Fatty Acyls	0.75	12.126	31407566.52	0.365	0.046%
Isoschaftoside	C26 H28 O14	52012-29-0	Flavonoids	1.72	6.449	30999903.54	0.360	0.046%
α-Linolenic acid	C18 H30 O2	463-40-1	Fatty Acyls	1.09	16.522	30326620.81	0.352	0.045%
Phthaldialdehyde	C8 H6 O2	643-79-8	Benzene and substituted derivatives	1.92	5.285	29772522.22	0.346	0.044%
Norcimifugin	C15 H16 O6	49624-66-0	Benzopyrans	0.73	8.733	29741203.57	0.345	0.044%
Senkyunolide A	C12 H16 O2	63038-10-8	Isobenzofurans	1.77	11.872	29653798.21	0.344	0.044%
DL-Tryptophan	C11 H12 N2 O2	153-94-6	Indoles and derivatives	1.69	4.605	29652312.37	0.344	0.044%
2,4-Xylidine	C8 H11 N	95-68-1	Benzene and substituted derivatives	2.91	9.148	28709730.02	0.333	0.042%
8-{3-Oxo-2-[(2E)-2-penten-1-yl]-1-cyclopenten-1-yl}octanoic acid	C18 H28 O3	NA	Fatty Acyls	0.55	11.417	28513900.7	0.331	0.042%
Atractylenolide II	C15 H20 O2	73069-14-4	Prenol lipids	1.43	6.677	28457314.18	0.331	0.042%
4-Isobutylbenzoic acid	C11 H14 O2	38861-88-0	Benzene and substituted derivatives	1.56	7.388	28229146.42	0.328	0.042%
Ursonic acid	C30 H46 O3	6246-46-4	Prenol lipids	1.37	13.533	27681802.27	0.322	0.041%
3-(1-hydroxyethyl)-2,3,6,7,8,8a-hexahydropyrrolo[1,2-a]pyrazine-1,4-dione	C9 H14 N2 O3	1006-94-8	Carboxylic acids and derivatives	2.04	1.872	27502054.76	0.319	0.041%

Pinolenic acid	C18 H30 O2	16833-54-8	Fatty Acyls	0.81	12.011	27205657.49	0.316	0.040%
Benzoic acid	C7 H6 O2	65-85-0	Benzene and substituted derivatives	3.03	4.356	26611648.05	0.309	0.039%
Nicotinic acid	C6 H5 N O2	59-67-6	Pyridines and derivatives	3.11	6.688	26294128.2	0.305	0.039%
Echinocystic acid	C30 H48 O4	510-30-5	Prenol lipids	1.23	12.262	26037160.71	0.302	0.038%
Betaxolol	C18 H29 N O3	63659-18-7	Phenols	0.68	8.892	24399163.53	0.283	0.036%
Nictoflorin	C27 H30 O15	17650-84-9	Flavonoids	1.63	6.895	24021850.84	0.279	0.035%
2-Adamantanone	C10 H14 O	700-58-3	Organooxygen compounds	1.78	5.141	24007199.06	0.279	0.035%
Ligustilide	C12 H14 O2	4431-01-0	Isobenzofurans	1.36	12	23532335.7	0.273	0.035%
Cetrimonium	C19 H41 N	6899-10-1	Organonitrogen compounds	0.76	12.649	23089407.47	0.268	0.034%
5-Hydroxy-1-tetralone	C10 H10 O2	28315-93-7	Tetralins	1.46	4.575	23022078.31	0.267	0.034%
Butyl 4-aminobenzoate	C11 H15 N O2	94-25-7	Benzene and substituted derivatives	1.65	9.464	22583578.71	0.262	0.033%
Kynurenic acid	C10 H7 N O3	492-27-3	Quinolines and derivatives	1.51	4.975	22449399.18	0.261	0.033%
Aflatoxin G2	C17 H14 O7	7241-98-7	Coumarins and derivatives	0.67	9.893	22237925.25	0.258	0.033%
(3aR,4aS,5R,7aS,8S,9aR)-5-Hydroxy-4a,8-dimethyl-3-methyleneoctahydroazuleno[6,5-b]furan-2,6(3H,4H)-dione	C15 H20 O4	NA	Prenol lipids	1.29	4.952	22113703.64	0.257	0.033%
7-Methyl-3-methylene-6-(3-oxobutyl)-3,3a,4,7,8,8a-hexahydro-2H-cyclohepta[b]furan-2-one	C15 H20 O3	NA	Prenol lipids	0.95	9.675	21720357.39	0.252	0.032%
β-Asarone	C12 H16 O3	5273-86-9	Phenol ethers	1.69	6.357	21332290.34	0.248	0.032%
(3β,5ξ,9ξ)-3,23-Dihydroxy-1-oxoolean-12-en-28-oic acid	C30 H46 O5	NA	Prenol lipids	1.25	10.226	20810062.42	0.242	0.031%
5,7,3'-Trihydroxy-6,4',5'-trimethoxyflavone	C18 H16 O8	NA	Flavonoids	0.86	10.348	20779929.86	0.241	0.031%
Genistein 4'-O-glucuronide	C21 H18 O11	245084-07-5	Isoflavonoids	0.97	7.31	20779583.3	0.241	0.031%
Isopropyl 4-Hydroxybenzoate	C10 H12 O3	4191-73-5	Benzene and substituted derivatives	1.87	3.668	20671623.96	0.240	0.031%
trans-3-Indoleacrylic acid	C11 H9 N O2	1204-06-4	Indoles and derivatives	1.78	5.716	19877253.37	0.231	0.029%
3',5,7-Trihydroxy-4'-methoxyflavanone	C16 H14 O6	NA	Flavonoids	0.87	10.065	19673342.72	0.229	0.029%
6-Hydroxy-8-methoxy-3-methyl-3,4-dihydro-1H-isochromen-1-one	C11 H12 O4	NA	Benzopyrans	1.51	4.726	19454856.76	0.226	0.029%
2,3,5,6-Tetramethylpyrazine	C8 H12 N2	1124-11-4	Diazines	1.91	4.95	19439115.5	0.226	0.029%
5,7-Dihydroxy-2-(4-hydroxy-3-methoxyphenyl)-4-oxo-4H-chromen-3-yl 6-O-(6-deoxy-α-L-mannopyranosyl)hexopyranoside	C28 H32 O16	604-80-8	Flavonoids	1.62	6.963	19342988.12	0.225	0.029%
Thymine	C5 H6 N2 O2	65-71-4	Diazines	2.76	2.093	18822009.51	0.219	0.028%
2-Aminooctadec-4-yne-1,3-diol	C18 H35 N O2	20256-56-8	Organonitrogen compounds	0.44	11.303	18560854.16	0.216	0.027%
6-Hydroxy-5a,9-dimethyl-3-methylene-3a,4,5,5a,6,7,9a,9b-octahydronaphtho[1,2-b]furan-2(3H)-one	C15 H20 O3	4290-13-5	Prenol lipids	0.8	8.897	18328480.75	0.213	0.027%
4-Methyl-5-thiazoleethanol	C6 H9 N O S	137-00-8	Azoles	2.02	1.725	17980511.3	0.209	0.027%

L-Asarinin	C20 H18 O6	133-03-9	Furanoid lignans	0.74	9.12	17536127.37	0.204	0.026%
18-β-Glycyrrhetinic acid	C30 H46 O4	1449-05-4	Prenol lipids	1.25	9.13	17376877.15	0.202	0.026%
Eriodictyol	C15 H12 O6	552-58-9	Flavonoids	0.79	8.957	17335898.93	0.201	0.026%
Skimmin	C15 H16 O8	93-39-0	Coumarins and derivatives	1.18	5.712	16966463.07	0.197	0.025%
Swertiamarin	C16 H22 O10	17388-39-5	Organooxygen compounds	1.06	2.832	16381124.54	0.190	0.024%
4-Hydroxybenzaldehyde	C7 H6 O2	123-08-0	Organooxygen compounds	3.29	1.349	16307828.82	0.189	0.024%
L-Isoleucine	C6 H13 N O2	NA	Carboxylic acids and derivatives	1.93	1.11	16189365.56	0.188	0.024%
Cantharidin	C10 H12 O4	56-25-7	Furofurans	1.43	5.234	16016059.12	0.186	0.024%
5-Phenylcyclohexane-1,3-dione	C12 H12 O2	493-72-1	Organooxygen compounds	1.45	9.796	15816423.47	0.184	0.023%
3,4-Dimethoxy-α-pyrrolidinopentiophenone	C17 H25 N O3	850442-84-1	Organooxygen compounds	0.68	7.96	15454368.42	0.180	0.023%
Oleoyl ethanolamide	C20 H39 N O2	111-58-0	Organonitrogen compounds	0.85	16.218	14743419.27	0.171	0.022%
Syringic acid	C9 H10 O5	530-57-4	Benzene and substituted derivatives	3.3	6.149	14605443.67	0.170	0.022%
Arachidonic acid	C20 H32 O2	506-32-1	Fatty Acyls	1.54	13.626	14398282.35	0.167	0.021%
Carvone	C10 H14 O	6485-40-1	Prenol lipids	1.98	10.888	14218989.7	0.165	0.021%
Palmitoyl ethanolamide	C18 H37 N O2	544-31-0	Carboximidic acids and derivatives	1.22	15.598	13224078.26	0.154	0.020%
6-O-Methylscutellarin	C22 H20 O12	31105-76-7	Flavonoids	2.24	7.487	12924323.93	0.150	0.019%
D-1,2,3,4-Tetrahydroisoquinoline-3-carboxylic acid	C10 H11 N O2	74163-81-8	Tetrahydroisoquinolines	2	2.475	12790627.31	0.149	0.019%
2'-O-Methyladenosine	C11 H15 N5 O4	42173-86-4	Purine nucleosides	1.12	1.591	12748428.85	0.148	0.019%
Apocynin	C9 H10 O3	498-02-2	Organooxygen compounds	2.09	5.528	12519177.97	0.145	0.018%
6-Amino-3-methyl-1-phenyl-1H-pyrazolo[3,4-b]pyridine-5-carboxamide	C14 H13 N5 O	NA	Azoles	-3.63	3.827	12506635.57	0.145	0.018%
Verrucarol	C15 H22 O4	2198-92-7	Prenol lipids	1.04	9.677	12491894.32	0.145	0.018%
4-amino-1-phenyl-6-(phenylimino)-1,2,5,6-tetrahydro-1,3,5-triazin-2-one	C15 H13 N5 O	NA	Benzene and substituted derivatives	-3.91	5.385	12201370.96	0.142	0.018%
7-[(6-Deoxy-α-L-mannopyranosyl)oxy]-5-hydroxy-2-(4-methoxyphenyl)-4-oxo-4H-chromen-3-yl β-D-glucopyranoside	C28 H32 O15	NA	Flavonoids	1.81	7.052	12057544.06	0.140	0.018%
Indole-3-acetyl-L-aspartic acid	C14 H14 N2 O5	2456-73-7	Carboxylic acids and derivatives	0.79	5.408	11528583.41	0.134	0.017%
Isoeugenol acetate	C12 H14 O3	93-29-8	Phenol esters	1.59	7.681	11526772.92	0.134	0.017%
Hypoxanthine	C5 H4 N4 O	68-94-0	Imidazopyrimidines	1.71	1.356	11312161.08	0.131	0.017%
Isosakuranin	C22 H24 O10	491-69-0	Flavonoids	1.23	8.556	11310180.11	0.131	0.017%
N-Acetyl-L-glutamate	C7 H11 N O5	1188-37-0	Carboxylic acids and derivatives	1.6	1.068	11129731.25	0.129	0.016%
Ancymidol	C15 H16 N2 O2	12771-68-5	Phenol ethers	1.05	10.575	10781485.04	0.125	0.016%
3,4-Dihydroxybenzaldehyde	C7 H6 O3	139-85-5	Organooxygen compounds	1.88	5.093	10730791.98	0.125	0.016%
Diosmetin	C16 H12 O6	520-34-3	Flavonoids	0.86	11.443	10244853.8	0.119	0.015%
Rilpivirine	C22 H18 N6	500287-72-9	Benzene and substituted derivatives	-2.27	8.651	10233566.86	0.119	0.015%
Diethyl phosphate	C4 H11 O4 P	66-76-2	Organic phosphoric acids and derivatives	1.56	1.376	10164469.84	0.118	0.015%
Aflatoxin B2	C17 H14 O6	7220-81-7	Coumarins and derivatives	0.55	10.658	10070327.05	0.117	0.015%

Shogaol	C17 H24 O3	555-66-8	Phenols	1.17	13.213	9745424.67	0.113	0.014%
4-Indolecarbaldehyde	C9 H7 N O	1074-86-6	Indoles and derivatives	1.7	7.608	8821151.279	0.102	0.013%
Isosteviol	C20 H30 O3	27975-19-5	Prenol lipids	0.87	12.347	8820487.647	0.102	0.013%
19-Norandrostenedione	C18 H24 O2	734-32-7	Steroids and steroid derivatives	1	10.87	8766106.506	0.102	0.013%
Kahweol	C20 H26 O3	6894-43-5	Naphthofurans	0.95	15.568	8747491.244	0.102	0.013%
Isomeranzin	C15 H16 O4	1088-17-1	Coumarins and derivatives	0.77	8.063	8440204.561	0.098	0.012%
Hydrolyzed fumonisin B1	C22 H47 N O5	145040-09-1	Organonitrogen compounds	1.21	10.598	8377981.828	0.097	0.012%
Zaleplon	C17 H15 N5 O	151319-34-5	Diazines	-3.41	8.574	8181579.896	0.095	0.012%
Vitexin rhamnoside	C27 H30 O14	64820-99-1	Flavonoids	1.28	6.762	7772019.745	0.090	0.011%
α -Linolenoyl ethanolamide	C20 H35 N O2	2387-23-8	Organonitrogen compounds	1.23	15.735	7389671.247	0.086	0.011%
Benzyladenine	C12 H11 N5	1214-39-7	Imidazopyrimidines	-4.83	1.443	7349343.13	0.085	0.011%
(3aR,8R,8aR,9aR)-8-Hydroxy-8a-methyl-3,5-bis(methylene)decahydronaphtho[2,3-b]furan-2(3H)-one	C15 H20 O3	NA	Prenol lipids	1.32	9.864	7281169.541	0.085	0.011%
Meperidine	C15 H21 N O2	57-42-1	Piperidines	0.99	7.933	7117168.069	0.083	0.011%
Isorhamnetin	C16 H12 O7	480-19-3	Flavonoids	1.17	8.392	7061775.768	0.082	0.010%
Cyclo(leucylprolyl)	C11 H18 N2 O2	5654-86-4	Carboxylic acids and derivatives	1.6	6.525	6990079.901	0.081	0.010%
Scopolin	C16 H18 O9	531-44-2	Coumarins and derivatives	1.37	4.875	6549601.485	0.076	0.010%
Eriocitrin	C27 H32 O15	13463-28-0	Flavonoids	1.98	6.521	6541942.175	0.076	0.010%
Ginkgolic acid (C13:0)	C20 H32 O3	20261-38-5	Benzene and substituted derivatives	0.79	11.398	6513257.399	0.076	0.010%
6''-O-Acetylglycitin	C24 H24 O11	134859-96-4	Isoflavonoids	1.61	10.109	6461702.824	0.075	0.010%
Levistilide A	C24 H28 O4	88182-33-6	Dihydrofurans	1.01	15.425	6268816.715	0.073	0.009%
Docosahexaenoic acid	C22 H32 O2	6217-54-5	Fatty Acyls	1.18	15.008	5841145.271	0.068	0.009%
(1S,4aR,5S)-5-[(3E)-5-Methoxy-3-methyl-5-oxo-3-penten-1-yl]-1,4a-dimethyl-6-methylenedecahydro-1-naphthalenecarboxylic acid	C21 H32 O4	NA	Prenol lipids	1.14	13.966	5821626.227	0.068	0.009%
Oleanonic acid	C30 H46 O3	17990-42-0	Prenol lipids	1.11	16.12	5732531.549	0.067	0.008%
Eicosatetraynoic acid	C20 H24 O2	1191-85-1	Fatty Acyls	0.41	12.396	5704015.374	0.066	0.008%
Kanosamine	C6 H13 N O5	576-44-3	Organooxygen compounds	1.62	0.806	5296061.014	0.062	0.008%
Polydatin	C20 H22 O8	27208-80-6	Stilbenes	1.07	10.571	5165678.637	0.060	0.008%
Indole-3-lactic acid	C11 H11 N O3	101312-07-6	Indoles and derivatives	1.75	6.275	5108846.137	0.059	0.008%
Tridemorph	C19 H39 N O	81412-43-3	Oxazinanes	1.04	19.324	4725674.604	0.055	0.007%
Aloesin	C19 H22 O9	30861-27-9	Organooxygen compounds	-4.71	5.21	4512272.171	0.052	0.007%
Naphyrone	C19 H23 N O	850352-53-3	Benzene and substituted derivatives	0.76	13.115	4451992.306	0.052	0.007%
Decanamide	C10 H21 N O	2319-29-1	Carboximidic acids and derivatives	1.36	11.407	4241764.942	0.049	0.006%
MDPBP	C15 H19 N O3	784985-33-7	Benzene and substituted derivatives	1.21	13.754	3656918.972	0.042	0.005%
[(3S)-3-{5-[4-(Dimethylamino)phenyl]-1,3,4-oxadiazol-2-yl}-1-pyrrolidinyl](3-pyridinyl)methanone	C20 H21 N5 O2	NA	Others	-2.73	8.614	3577198.973	0.042	0.005%

Phloretin	C15 H14 O5	60-82-2	Linear 1,3-diarylpropanoids	1	8.579	3212802.887	0.037	0.005%
Palmitoleic acid	C16 H30 O2	373-49-9	Fatty Acyls	1.02	15.21	2957366.835	0.034	0.004%
Muscone	C16 H30 O	541-91-3	Organooxygen compounds	1.1	17.293	2771610.81	0.032	0.004%

Table S2. Phytochemical profile of ZLE characterized by UPLC-Q-Exactive HF-MS in negative ion mode (ESI-).

Name	Formula	CAS_num	Class	Annot. DeltaMass (ppm)	RT (min)	Area	Concentration (μg/mL)	Relative Contents (%)
Geniposide	C17 H24 O10	24512-63-8	Prenol lipids	1.95	6.044	19706191709	1719.285046	24.823%
Genipin 1-O-β-D-gentiobioside	C23 H34 O15	29307-60-6	Prenol lipids	2.34	5.572	4045064227	352.9153953	5.095%
Methyl 1-(hexopyranosyloxy)-5-hydroxy-7-(hydroxymethyl)-1,4a,5,7a-tetrahydro-cyclopenta[c]pyran-4-carboxylate	C17 H24 O11	NA	Prenol lipids	1.83	4.562	3960339626	345.5235185	4.989%
D-(-)-Quinic acid	C7 H12 O6	77-95-2	Organooxygen compounds	-2.13	1.163	3386613660	295.4682624	4.266%
Ethyl 4-{[3-(4-methoxyphenyl)-1,2,4-oxadiazol-5-yl]methyl}-1-piperazinecarboxylate	C17 H22 N4 O4	NA	Azoles	-2.4	4.664	3287673199	286.8361098	4.141%
Citric acid	C6 H8 O7	77-92-9	Carboxylic acids and derivatives	-2.13	1.438	2708866935	236.3376182	3.412%
Ethyl 4-{[(2E)-2-(2,4-dimethoxybenzylidene)hydrazino](oxo)acetyl}-1-piperazinecarboxylate	C18 H24 N4 O6	469898-99-5	Carboxylic acids and derivatives	-1.57	5.261	2170942474	189.4058978	2.735%
Shanzhiside	C16 H24 O11	29836-27-9	Prenol lipids	1.95	3.018	2125827206	185.4697741	2.678%
Ethyl 4-({[1-(ethylcarbamoyl)-3-oxo-2-piperazinyl]acetyl}amino)benzoate	C18 H24 N4 O5	NA	Benzene and substituted derivatives	-1.77	6.398	1990143853	173.6319537	2.507%
(1S,3S)-3-Acetyl-3,5,12-trihydroxy-10-methoxy-6,11-dioxo-1,2,3,4,6,11-hexahydro-1-tetracenyl 2,3,6-trideoxy-3-({[2-(2-nitro-1H-imidazol-1-yl)ethoxy]methyl}amino)-alpha-L-lyxo-hexopyranoside	C33 H36 N4 O13	NA	Others	0.54	7.528	1910864080	166.7151161	2.407%
Naringin	C27 H32 O14	10236-47-2	Flavonoids	2.02	7.273	1847434617	161.1811535	2.327%
Hesperidin	C28 H34 O15	520-26-3	Flavonoids	2	7.525	1821543179	158.9222309	2.295%
(3beta)-16,18-Dioxo-18,20-epoxylanosta-9(11),25-dien-3-yl 6-O-sulfo-beta-D-glucopyranosyl-(1->4)-6-deoxy-alpha-D-galactopyranosyl-(1->2)-4-O-sulfo-beta-D-xylopyranoside	C47 H72 O23 S2	NA	Organooxygen compounds	1.32	7.374	1620994345	141.4251611	2.042%
Ethyl 1'-(2-pyrimidinyl)-3a,4,8,8a-tetrahydrospiro[1,3-dioxepino[5,6-d][1,2]oxazole-6,4'-piperidine]-3-carboxylate	C17 H22 N4 O5	NA	Others	-1.93	3.028	1490360930	130.0279272	1.877%

N-{2-[4-(1,1-Dioxido-3-oxo-1,2-thiazolidin-5-yl)phenyl]-1-[4-(4-phenylbutyl)-1H-imidazol-2-yl]ethyl}-3-(trifluoromethyl)benzenesulfonamide	C31 H31 F3 N4 O5 S2	NA	Benzene and substituted derivatives	2.33	7.8	1341647982	117.0533275	1.690%
4,5-Dicaffeoylquinic acid	C25 H24 O12	14534-61-3	Organooxygen compounds	1.68	7.646	1294379629	112.9293561	1.630%
(1R)-1,5-Anhydro-6-deoxy-2-O-(6-deoxy-a-L-mannopyranosyl)-1-[5,7-dihydroxy-2-(4-hydroxyphenyl)-4-oxo-4H-chromen-6-yl]-L-ribo-hex-3-ulose	C27 H28 O13	NA	Flavonoids	1.8	8.164	1255134002	109.5053348	1.581%
sinapoylglucose	C17 H22 O10	78185-48-5	Cinnamic acids and derivatives	1.71	6.02	1125694892	98.21229914	1.418%
Lyoniside	C27 H36 O12	34425-25-7	Lignan glycosides	2.07	7.936	981735122.2	85.65239493	1.237%
Chlorogenic acid	C16 H18 O9	202650-88-2	Organooxygen compounds	1.48	5.55	950168494.9	82.89833514	1.197%
[4-(3,5-Dinitrobenzoyl)-1-piperazinyl](2-furyl)methanone	C16 H14 N4 O7	487018-63-3	Benzene and substituted derivatives	-2.25	5.41	889190980.3	77.57829509	1.120%
Rutin	C27 H30 O16	153-18-4	Flavonoids	2.33	6.836	779362654.1	67.9962205	0.982%
3,5-Dicaffeoylquinic acid	C25 H24 O12	2450-53-5	Organooxygen compounds	1.41	7.501	777715448.5	67.85250851	0.980%
2-C-methylerythritol 4-phosphate	C5 H13 O7 P	206440-72-4	Organic phosphoric acids and derivatives	0.15	1.124	764159120.3	66.66977404	0.963%
dimethyl citrate	C8 H12 O7	53798-97-3	Carboxylic acids and derivatives	-0.79	4.598	709824950.7	61.92933882	0.894%
N,N'-1,2-Propanediylbis(2-nitrobenzamide)	C17 H16 N4 O6	NA	Benzene and substituted derivatives	-2.13	5.456	673649627.8	58.77318908	0.849%
(15Z)-9,12,13-Trihydroxy-15-octadecenoic acid	C18 H34 O5	NA	Fatty Acyls	1.54	10.308	640540972.8	55.88459365	0.807%
AF4878000	C11 H12 O3	94-02-0	Organooxygen compounds	-2.24	10.709	639186244.9	55.76639915	0.805%
4,7-Dinitro-2-oxo-5-phenyl-2,3,4,5-tetrahydro-1H-1,4-benzodiazepin-5-yl acetate	C17 H14 N4 O7	62658-10-0	Benzodiazepines	-1.3	5.929	623573151.7	54.40422029	0.785%
Methyl 1-(hexopyranosyloxy)-4a-hydroxy-7-methyl-5-oxo-1,4a,5,6,7,7a-hexahydrocyclopenta[c]pyran-4-carboxylate	C17 H24 O11	50816-24-5	Prenol lipids	1.73	4.861	608472430.2	53.08674379	0.766%
Diosmetin-7-O-β-D-glucopyranoside	C22 H22 O11	20126-59-4	Flavonoids	2.36	6.551	498960995	43.53231664	0.629%
geniposidic acid	C16 H22 O10	27741-01-1	Prenol lipids	2.04	2.302	488127976.9	42.58717989	0.615%
α,α-Trehalose	C12 H22 O11	99-20-7	Organooxygen compounds	1.46	1.127	481518797.9	42.01055592	0.607%
D-(-)-Mannitol	C6 H14 O6	45007-61-2	Organooxygen compounds	-2.64	1.114	437199472.7	38.14387512	0.551%
Asiatic acid	C30 H48 O5	464-92-6	Prenol lipids	1.89	12.19	422924728.1	36.89846173	0.533%
Citramalic acid	C5 H8 O5	490-83-5	Fatty Acyls	-4.98	1.675	413968184.1	36.11703971	0.521%
Quercetin-3β-D-glucoside	C21 H20 O12	482-35-9	Flavonoids	2.09	7.07	405713380	35.39684164	0.511%
6-O-Pentopyranosyl-1-O-[(2,6,6-trimethyl-1-cyclohexen-1-yl)carbonyl]-β-D-glucopyranose	C21 H34 O11	NA	Organooxygen compounds	1.83	8.024	404765332.4	35.31412835	0.510%
trans-Aconitic acid	C6 H6 O6	4023-65-8	Carboxylic acids and derivatives	-3.39	1.571	403824820.4	35.2320725	0.509%
Apigenin-7-O-β-D-glucoside	C21 H20 O10	578-74-5	Flavonoids	1.58	7.56	402057312.9	35.07786466	0.506%
Cynaroside	C21 H20 O11	5373-11-5	Flavonoids	2.1	7.085	372967264.6	32.53987631	0.470%
Arjungenin	C30 H48 O6	58880-25-4	Prenol lipids	2.09	11.226	364164166.9	31.77184185	0.459%
D-(+)-Galactose	C6 H12 O6	39392-63-7	Organooxygen compounds	-2.85	1.115	353541885.3	30.845091	0.445%

Luteolin	C15 H10 O6	491-70-3	Flavonoids	1.52	9.312	324472720.6	28.30892466	0.409%
Vicenin II	C27 H30 O15	23666-13-9	Flavonoids	1.73	6.029	306108077	26.70668423	0.386%
Gallic acid	C7 H6 O5	149-91-7	Benzene and substituted derivatives	-3.58	2.076	303580534.1	26.48616639	0.382%
Cabotegravir	C19 H17 F2 N3 O5	1051375-10-0	Pyridines and derivatives	-3.16	1.175	289553650.1	25.26237784	0.365%
Corchorifatty acid F	C18 H32 O5	95341-44-9	Fatty Acyls	1.59	9.951	288894929	25.2049071	0.364%
3-Butene-1,2,3-tricarboxylic acid	C7 H8 O6	26326-05-6	Carboxylic acids and derivatives	-2.45	1.805	282154926.6	24.61686932	0.355%
5-Dehydroquinic acid	C7 H10 O6	10534-44-8	Hydroxy acids and derivatives	-2.3	2.16	265258914.5	23.14276101	0.334%
Perillic acid	C10 H14 O2	7694-45-3	Prenol lipids	-4.39	4.666	263950607.6	23.02861655	0.332%
Quercetin 3-O-β-D-Glucuronide	C21 H18 O13	22688-79-5	Flavonoids	1.73	7.056	223546924.6	19.5035596	0.282%
Caffeic acid	C9 H8 O4	331-39-5	Cinnamic acids and derivatives	-3.43	6.069	197476530.1	17.22902376	0.249%
Poncirin	C28 H34 O14	14941-08-3	Flavonoids	2.32	8.857	178594851.8	15.58167415	0.225%
Genistein	C15 H10 O5	446-72-0	Isoflavonoids	1.11	10.101	177638810.2	15.49826342	0.224%
(+/-)9,10-dihydroxy-12Z-octadecenoic acid	C18 H34 O4	2345-28-3	Fatty Acyls	1.47	12.43	172852177	15.08064914	0.218%
2-Isopropylmalic acid	C7 H12 O5	3237-44-3	Fatty Acyls	-4.34	5.468	171947443.1	15.00171479	0.217%
Azelaic acid	C9 H16 O4	123-99-9	Fatty Acyls	-2.59	7.891	163532254.5	14.26752382	0.206%
Crocin	C44 H64 O24	42553-65-1	Prenol lipids	2.4	9.573	133423276.8	11.64063803	0.168%
3,4-Dihydroxyphenylacetic acid	C8 H8 O4	102-32-9	Phenols	-4.14	6	129168157.6	11.26939619	0.163%
Homoorientin	C21 H20 O11	4261-42-1	Flavonoids	1.77	6.459	111559666.4	9.733126976	0.141%
4-(4-Hydroxyphenyl)-2-butanyl 6-O-[(4ξ)-α-L-threo-pentofuranosyl]-β-D-glucopyranoside	C21 H32 O11	NA	Fatty Acyls	1.58	7.807	109631975.5	9.564943788	0.138%
1,6-Bis-O-(3,4,5-trihydroxybenzoyl)hexopyranose	C20 H20 O14	23363-08-8	Tannins	1.52	3.09	108147735.8	9.435449914	0.136%
(3R,5R)-1,3,5-Trihydroxy-4-{[(2E)-3-(4-hydroxy-3-methoxyphenyl)-2-propenoyl]oxy}cyclohexanecarboxylic acid	C17 H20 O9	NA	Organooxygen compounds	1.26	6.488	103833749.3	9.05907215	0.131%
2,6-Bis[(2-hydroxyethyl)amino]-3-nitrobenzonitrile	C11 H14 N4 O4	NA	Benzene and substituted derivatives	-3.57	1.744	103190962.7	9.002991634	0.130%
Narcissoside	C28 H32 O16	604-80-8	Flavonoids	2.05	7.273	101506533.9	8.856032075	0.128%
Lonicerin	C27 H30 O15	NA	Flavonoids	2.18	6.821	100097828	8.733128214	0.126%
Linoelaidic acid	C18 H32 O2	506-21-8	Fatty Acyls	1.34	17.69	99112414.19	8.64715487	0.125%
Glycitein	C16 H12 O5	40957-83-3	Isoflavonoids	1.48	11.582	99021642.98	8.639235451	0.125%
Luteolin 7-glucuronide	C21 H18 O12	29741-10-4	Flavonoids	2.32	7.107	93142303.94	8.126287042	0.117%
2,3-Dihydro-1-benzofuran-2-carboxylic acid	C9 H8 O3	1914-60-9	Coumarans	-4.25	6.956	91234203.9	7.959813077	0.115%
Oleanolic acid	C30 H48 O3	508-02-1	Prenol lipids	1.77	16.983	89572598.82	7.814844795	0.113%
Gentiopicrin	C16 H20 O9	20831-76-9	Organooxygen compounds	1.73	5.981	87724553.52	7.653610361	0.111%
8-O-Acetylharpagide	C17 H26 O11	6926-14-3	Prenol lipids	1.56	2.347	87222595	7.609816523	0.110%
Mussaenosidic acid	C16 H24 O10	82451-22-7	Prenol lipids	1.13	4.651	86221641.49	7.522487402	0.109%
2,2'-Methylenebis(4-methyl-6-tert-butylphenol)	C23 H32 O2	119-47-1	Benzene and substituted derivatives	0.88	17.306	82368869.54	7.186348725	0.104%

Methyl gallate	C8 H8 O5	99-24-1	Benzene and substituted derivatives	-2.77	9.433	79997402.56	6.97944788	0.101%
cis-Aconitic acid	C6 H6 O6	585-84-2	Carboxylic acids and derivatives	-3.53	4.422	79216251.09	6.911295593	0.100%
16-Hydroxyhexadecanoic acid	C16 H32 O3	506-13-8	Fatty Acyls	1.57	16.408	78512756.14	6.849918521	0.099%
(+/-)12(13)-DiHOME	C18 H34 O4	263399-35-5	Fatty Acyls	1.56	12.313	74711561.02	6.518279713	0.094%
D-Glucono-1,5-lactone	C6 H10 O6	90-80-2	Organooxygen compounds	-1.43	1.602	69431282.46	6.057596893	0.087%
Astragalin	C21 H20 O11	480-10-4	Flavonoids	1.71	7.438	67444185.53	5.884230771	0.085%
1-[4,5-Dihydroxy-6-(hydroxymethyl)-3-[(E)-3-(4-hydroxyphenyl)prop-2-enoyl]oxyoxan-2-yl]oxy-7-hydroxy-7-methyl-4a,5,6,7a-tetrahydro-1H-cyclopenta[c]pyran-4-carboxylic acid	C25 H30 O12	NA	Prenol lipids	2.05	6.752	65502427.49	5.714820283	0.083%
Kaempferol-3-O-rutinoside	C27 H30 O15	17650-84-9	Flavonoids	2.25	7.189	63088340.74	5.504201036	0.079%
3,5-Dihydroxy-2-(4-hydroxyphenyl)-4-oxo-3,4-dihydro-2H-chromen-7-yl hexopyranoside	C21 H22 O11	NA	Flavonoids	1.25	7.068	62402226.94	5.444340399	0.079%
Ferulic acid	C10 H10 O4	1135-24-6	Cinnamic acids and derivatives	0.46	8.973	58375800.42	5.093051067	0.074%
α-Eleostearic acid	C18 H30 O2	544-73-0	Fatty Acyls	1.04	16.421	55609309.31	4.851685974	0.070%
Quercetin	C15 H10 O7	117-39-5	Flavonoids	1.19	9.404	55136730.68	4.810455412	0.069%
L-Phenylalanine	C9 H11 N O2	150-30-1	Carboxylic acids and derivatives	-3.89	2.531	52520416.72	4.582192664	0.066%
Scutellarin	C21 H18 O12	27740-01-8	Flavonoids	2.17	7.457	51243825.89	4.470815308	0.065%
(3β,5ξ,9ξ)-3,23-Dihydroxy-1-oxoolean-12-en-28-oic acid	C30 H46 O5	NA	Prenol lipids	1.62	12.476	50834907.35	4.435138829	0.064%
Naringenin	C15 H12 O5	480-41-1	Flavonoids	1.24	10.13	50287710.19	4.387398103	0.063%
Eriodictyol	C15 H12 O6	552-58-9	Flavonoids	1.72	9.249	48053359.76	4.192460119	0.061%
5,7-Dihydroxy-2-(3-hydroxy-4-methoxyphenyl)chroman-4-one	C16 H14 O6	520-33-2	Flavonoids	1.2	10.363	46915136.66	4.093154785	0.059%
Uridine	C9 H12 N2 O6	58-96-8	Pyrimidine nucleosides	0.68	1.57	45685056.42	3.985835289	0.058%
Dodecyl sulfate	C12 H26 O4 S	151-41-7	Organic sulfuric acids and derivatives	1.32	14.693	44296117.33	3.864656005	0.056%
Kaempferol 3-glucorhamnoside	C27 H30 O15	40437-72-7	Flavonoids	2.18	7.064	41475751.42	3.618590555	0.052%
2-[3,8-Dihydroxy-8-(hydroxymethyl)-3-methyl-2-oxodecahydro-5-azulenyl]-2-propanyl hexopyranoside	C21 H36 O10	NA	Prenol lipids	1.89	8.862	39868729.16	3.478384401	0.050%
Baicalin	C21 H18 O11	21967-41-9	Flavonoids	1.97	7.574	39721948.86	3.465578417	0.050%
Grosvenorine	C33 H40 O19	156980-60-8	Flavonoids	2.83	6.58	37535269.97	3.274799581	0.047%
Sibiricose A1	C23 H32 O15	139726-40-2	Cinnamic acids and derivatives	1.6	6.141	37342708.92	3.257999413	0.047%
Bilobalide	C15 H18 O8	33570-04-6	Prenol lipids	1.27	5.606	35709721.36	3.115527893	0.045%
Chrysosplenetin B	C19 H18 O8	603-56-5	Flavonoids	1.24	11.667	34969391.65	3.050937138	0.044%
Vicenin III	C26 H28 O14	59914-91-9	Flavonoids	1.83	6.38	33708716.6	2.940948369	0.042%
2-(8-Hydroxy-4a,8-dimethyldecahydro-2-naphthalenyl)acrylic acid	C15 H24 O3	4586-68-9	Prenol lipids	1.42	11.442	33097257.22	2.887601027	0.042%
parsaclisib	C20 H22 Cl F N6 O2	1426698-88-5	Pyrrolidines	1.84	1.387	32874797.17	2.868192292	0.041%

Ethyl-β-D-glucuronide	C8 H14 O7	17685-04-0	Organooxygen compounds	-0.39	1.595	31872831.9	2.7807749	0.040%
Guanosine	C10 H13 N5 O5	118-00-3	Purine nucleosides	1.69	1.644	30934179.79	2.698881322	0.039%
Secoisolariciresinol diglucoside	C32 H46 O16	148244-82-0	Lignan glycosides	2.53	7.089	30886736	2.694742044	0.039%
N-Acetyl-L-phenylalanine	C11 H13 N O3	2018-61-3	Carboxylic acids and derivatives	-1.28	6.903	29884310.55	2.607284502	0.038%
DL-4-Hydroxyphenyllactic acid	C9 H10 O4	23508-35-2	Phenylpropanoic acids	-0.69	4.859	27713828.96	2.417918815	0.035%
Calcium pantothenate	C9 H17 N O5	137-08-6	Carboxylic acids and derivatives	-0.99	3.046	27229337.3	2.375648889	0.034%
L-Tyrosine	C9 H11 N O3	556-03-6	Carboxylic acids and derivatives	-2.93	1.608	26661113.2	2.326073648	0.034%
Jaceosidin	C17 H14 O7	18085-97-7	Flavonoids	1.68	10.192	26372630.92	2.300904743	0.033%
3-tert-Butyladipic acid	C10 H18 O4	10347-88-3	Fatty Acyls	-1.49	9.116	23032689.68	2.00950846	0.029%
Rosmarinic acid	C18 H16 O8	20283-92-5	Cinnamic acids and derivatives	1.35	7.855	22262201.69	1.942286517	0.028%
5,7,3'-Trihydroxy-6,4',5'-trimethoxyflavone	C18 H16 O8	NA	Flavonoids	1.86	10.647	21960455.69	1.915960407	0.028%
18-β-Glycyrrhetinic acid	C30 H46 O4	1449-05-4	Prenol lipids	1.43	14.973	20674239.02	1.803743235	0.026%
3',5'-Dimethoxy-4'-hydroxyacetophenone	C10 H12 O4	2478-38-8	Organooxygen compounds	-2.25	7.729	19009297.08	1.658483825	0.024%
6-Gingerol	C17 H26 O4	23513-14-6	Phenols	1.74	11.75	18824554.63	1.642365798	0.024%
3,4-Dihydroxyphenylpropionic acid	C9 H10 O4	1078-61-1	Phenylpropanoic acids	-2.87	8.361	18387725.65	1.604254247	0.023%
[7-(Diethylcarbamoyl)-1,5-dinitro-3-azabicyclo[3.3.1]non-6-en-3-yl]acetic acid	C15 H22 N4 O7	NA	Carboxylic acids and derivatives	-1.85	1.632	18100229.5	1.579171378	0.023%
Suberic acid	C8 H14 O4	505-48-6	Fatty Acyls	-3.46	6.961	17945731.84	1.565692085	0.023%
Crocin II	C38 H54 O19	55750-84-0	Prenol lipids	2.68	9.965	17896298.99	1.56137927	0.023%
13,14-dihydro Prostaglandin F1α	C20 H38 O5	20592-20-5	Fatty Acyls	1.54	11.873	17315434.01	1.510701164	0.022%
2-[(2S,4aR,8aS)-2-Hydroxy-4a-methyl-8-methylenedecahydro-2-naphthalenyl]acrylic acid	C15 H22 O3	NA	Prenol lipids	1.57	11.897	15954905.35	1.392000575	0.020%
(2'R,3R,4'R,4a'R,5S,8a'S)-5-(3-Furyl)-4'-hydroxy-4a',5'-bis(hydroxymethyl)-2'-methyl-3',4,4',4a',5,7',8',8a'-octahydro-2'H-spiro[furan-3,1'-naphthalen]-2-one	C20 H26 O6	NA	Prenol lipids	1.86	8.039	14731122.06	1.285230462	0.019%
1-(β-D-Glucopyranosyloxy)-7-methyl-1,4a,5,6,7,7a-hexahydrocyclopenta[c]pyran-4-carboxylic acid	C16 H24 O9	88668-99-9	Prenol lipids	2.71	6.726	14564840.79	1.270723097	0.018%
Chikusetsu saponin IVa	C42 H66 O14	51415-02-2	Prenol lipids	2.93	10.407	14055055.11	1.226246371	0.018%
3-Hydroxy-3-(methoxycarbonyl)pentanedioic acid	C7 H10 O7	26163-65-5	Carboxylic acids and derivatives	-1.44	2.215	13360935.05	1.16568722	0.017%
(±)-Abscisic acid	C15 H20 O4	14375-45-2	Prenol lipids	1.36	9.046	12956435.15	1.13039625	0.016%
Octyl gallate	C15 H22 O5	1034-01-1	Benzene and substituted derivatives	1.15	6.257	12680266.35	1.106301645	0.016%
Isorhamnetin	C16 H12 O7	480-19-3	Flavonoids	1.54	10.973	12307753.65	1.073801428	0.016%
Diosmin	C28 H32 O15	520-27-4	Flavonoids	1.62	7.364	12267880.65	1.070322671	0.015%
(-)-Camphanic acid	C10 H14 O4	13429-83-9	Lactones	-1.58	6.321	11847577.68	1.033652948	0.015%
N-Acetyl-DL-valine	C7 H13 N O3	3067-19-4	Carboxylic acids and derivatives	-4.45	4.744	11519342.35	1.00501575	0.015%
2-AMINO-3-(2-CHLORO-PHENYL)-PROPIONIC ACID	C9 H10 Cl N O2	14091-11-3	The internal standard	-1.02	4.809	22923705.12	1	0.000%
3-Methoxy-5,7,3',4'-tetrahydroxy-flavone	C16 H12 O7	1486-70-0	Flavonoids	1.74	10.375	11393791.75	0.994061971	0.014%

Bruceine A	C26 H34 O11	25514-31-2	Prenol lipids	1.61	8.136	11354685.24	0.990650088	0.014%
Eupatilin	C18 H16 O7	22368-21-4	Flavonoids	1.18	11.222	11236777.58	0.980363123	0.014%
Ethyl alpha-D-glucopyranoside	C8 H16 O6	19467-01-7	Organooxygen compounds	0.37	1.673	11157737.58	0.973467205	0.014%
N4-(2,1,3-Benzoxadiazol-5-yl)morpholine-4-carboxamide	C11 H12 N4 O3	NA	Benzoxadiazoles	-4.55	2.123	10680973.79	0.931871504	0.013%
3,3'-Diisopropyl-6,6'-dimethyl-2,2',5,5'-biphenyltetrol	C20 H26 O4	NA	Benzene and substituted derivatives	1.32	11.426	9519200.796	0.830511538	0.012%
D-(+)-Tryptophan	C11 H12 N2 O2	153-94-6	Indoles and derivatives	-2.12	4.84	9209962.008	0.803531712	0.012%
2-Aminooctanedioic acid	C8 H15 N O4	19641-59-9	Carboxylic acids and derivatives	-2.65	1.8	8914157.339	0.777723958	0.011%
3',6-Disinapoyl sucrose	C34 H42 O19	139891-98-8	Cinnamic acids and derivatives	2.64	6.818	8628773.08	0.752825343	0.011%
Prostaglandin F1 α	C20 H36 O5	745-62-0	Fatty Acyls	1.53	11.47	8534278.091	0.744581039	0.011%
(R)-3-Hydroxy myristic acid	C14 H28 O3	1961-72-4	Fatty Acyls	0.29	13.739	8525238.95	0.743792411	0.011%
2-Hydroxymyristic acid	C14 H28 O3	NA	Fatty Acyls	0.04	12.863	8309118.93	0.724936819	0.010%
Casticin	C19 H18 O8	479-91-4	Flavonoids	1.48	11.474	7734177.865	0.67477555	0.010%
Hexadecanedioic acid	C16 H30 O4	505-54-4	Fatty Acyls	1.25	10.879	7705597.809	0.672282056	0.010%
1,2,3,4-Tetrahydro-7-hydroxy-1-(4-hydroxy-3-methoxyphenyl)-6-methoxy-2,3-naphthalenedimethanol	C20 H24 O6	548-29-8	Lignans, neolignans and related compounds	1.81	13.036	7084136.815	0.618062113	0.009%
1-(3,4-Dihydroxyphenyl)-6,7-dihydroxy-N,N'-bis[2-(4-hydroxyphenyl)ethyl]-2,3-naphthalenedicarboxamide	C34 H30 N2 O8	130508-46-2	Arylnaphthalene lignans	2.79	1.39	6828600.938	0.595767648	0.009%
Coumafuryl	C17 H14 O5	117-52-2	Coumarins and derivatives	1.22	12.412	6094601.73	0.531729203	0.008%
2-Cyclopentylphenol	C11 H14 O	1518-84-9	Phenols	-4.55	10.931	6010201.644	0.524365639	0.008%
Melittoside	C21 H32 O15	19467-03-9	Prenol lipids	1.22	4.744	5801206.77	0.506131687	0.007%
Dodecanedioic acid	C12 H22 O4	693-23-2	Fatty Acyls	-0.07	10.678	5257590.872	0.458703412	0.007%
1,9b-Dihydroxy-6,6,9a-trimethyl-5,5a,6,7,8,9,9a,9b-octahydronaphtho[1,2-c]furan-3(1H)-one	C15 H22 O4	NA	Naphthofurans	1.72	10.482	5230498.622	0.456339723	0.007%
Tretinoin	C20 H28 O2	302-79-4	Prenol lipids	0.73	13.644	5052842.968	0.44083999	0.006%
N-[(1S,3aS,5S,7aR)-5-Hydroxy-7a-{3-[4-(2-methoxyphenyl)-1-piperazinyl]-3-oxopropyl}-3,3,5-trimethyloctahydro-1H-inden-1-yl]nicotinamide	C32 H44 N4 O4	NA	Others	-0.44	11.033	5006262.452	0.436776029	0.006%
Pectolinarigenin	C17 H14 O6	520-12-7	Flavonoids	1.7	11.991	4761878.437	0.415454518	0.006%
3,5-di-tert-Butyl-4-hydroxybenzyl alcohol	C15 H24 O2	88-26-6	Benzene and substituted derivatives	0.05	12.951	4552240.983	0.397164504	0.006%
Usnic acid	C18 H16 O7	125-46-2	Benzene and substituted derivatives	1.36	12.149	4379488.198	0.382092526	0.006%
Crocetin	C20 H24 O4	27876-94-4	Prenol lipids	1.41	12	4184728.45	0.36510053	0.005%
(1S,4aR,5S)-5-[(3E)-5-Methoxy-3-methyl-5-oxo-3-penten-1-yl]-1,4a-dimethyl-6-methylenedecahydro-1-naphthalenecarboxylic acid	C21 H32 O4	NA	Prenol lipids	1.48	14.271	3809190.221	0.332336348	0.005%

(3β,5ξ,9ξ,18ξ)-28-Hydroxy-28-oxoolean-12-en-3-yl 6-deoxy-α-L-mannopyranosyl-(1->3)-[β-D-glucopyranosyl-(1->2)]-β-D-glucopyranosiduronic acid	C48 H76 O18	NA	Prenol lipids	2.23	10.13	3407219.802	0.297266064	0.004%
Curcumin	C21 H20 O6	458-37-7	Diarylheptanoids	0.92	12.608	3004683.988	0.262146453	0.004%
4-tert-Amylphenol	C11 H16 O	80-46-6	Benzene and substituted derivatives	-2.63	11.323	2595663.103	0.226461045	0.003%
6-Amino-3-methyl-4-phenyl-1,4-dihydropyrano[2,3-c]pyrazole-5-carbonitrile	C14 H12 N4 O	NA	Benzene and substituted derivatives	-4.27	13.54	2419540.294	0.211095046	0.003%
(-)-Spiculisporic acid	C17 H28 O6	65759-98-0	Carboxylic acids and derivatives	1.37	12.468	2259179.495	0.197104219	0.003%
Androsin	C15 H20 O8	531-28-2	Organooxygen compounds	1.8	8.292	2155937.601	0.188096784	0.003%
2,5-di-tert-Butylhydroquinone	C14 H22 O2	88-58-4	Benzene and substituted derivatives	-0.8	11.749	1883438.868	0.164322378	0.002%
